# Investigating the effect of bacteriophages on bacterial FtsZ localisation

**DOI:** 10.3389/fcimb.2022.863712

**Published:** 2022-07-29

**Authors:** Gurneet K. Dhanoa, Inbar Kushnir, Udi Qimron, David I. Roper, Antonia P. Sagona

**Affiliations:** ^1^ School of Life Sciences, University of Warwick, Coventry, United Kingdom; ^2^ Department of Clinical Microbiology and Immunology, Sackler School of Medicine, Tel Aviv University, Tel Aviv, Israel

**Keywords:** bacteriophages, FtsZ, microscopy, inhibitors, filamentation, human cells, cell division

## Abstract

*Escherichia coli* is one of the most common Gram-negative pathogens and is responsible for infection leading to neonatal meningitis and sepsis. The FtsZ protein is a bacterial tubulin homolog required for cell division in most species, including *E. coli*. Several agents that block cell division have been shown to mislocalise FtsZ, including the bacteriophage λ-encoded Kil peptide, resulting in defective cell division and a filamentous phenotype, making FtsZ an attractive target for antimicrobials. In this study, we have used an *in vitro* meningitis model system for studying the effect of bacteriophages on FtsZ using fluorescent *E. coli* EV36/FtsZ-mCherry and K12/FtsZ-mNeon strains. We show localisation of FtsZ to the bacterial cell midbody as a single ring during normal growth conditions, and mislocalisation of FtsZ producing filamentous multi-ringed bacterial cells upon addition of the known inhibitor Kil peptide. We also show that when bacteriophages K1F-GFP and T7-mCherry were applied to their respective host strains, these phages can inhibit FtsZ and block bacterial cell division leading to a filamentous multi-ringed phenotype, potentially delaying lysis and increasing progeny number. This occurs in the exponential growth phase, as actively dividing hosts are needed. We present that ZapA protein is needed for phage inhibition by showing a phenotype recovery with a ZapA mutant strain, and we show that FtsI protein is also mislocalised upon phage infection. Finally, we show that the T7 peptide gp0.4 is responsible for the inhibition of FtsZ in K12 strains by observing a phenotype recovery with a T7Δ0.4 mutant.

## 1 Introduction

Antimicrobial resistance is a growing problem worldwide, and infections caused by Gram-negative bacteria are particularly concerning, as these organisms are highly efficient at acquiring genes for antibiotic drug resistance (Peleg and Hooper, 2010; ECDC, 2019), in addition to permeability issues presented by the outer membrane. This has led to the need for novel antibacterial agents and model systems in which to test them. A potential target for new molecules could be the bacterial cell division machinery, as this is essential for bacterial propagation and survival and is directly related to bacterial cell wall biosynthesis, which is a validated target for natural products and semisynthetic clinically used antibiotics (Araya et al., 2019).

*Escherichia coli* is one of the most common Gram-negative pathogens and is responsible for many different diseases. For example, *E. coli* O18:K1:H7 is responsible for secondary infections in burn patients, neonatal meningitis (Baud and Aujard, 2013), and sepsis, which can rapidly lead to shock and mortality (Busch et al., 2000). Approximately 80% of the *E. coli* strains that are able to cause meningitis are of the K1 capsule type (Kaper et al., 2004). The K1 antigen produced by these strains makes up a thick polysaccharide capsule, which aids pathogenicity by immune system evasion, giving bacterial cells the ability to cross certain barriers (such as the blood–brain barrier) and defend against certain bacteriophages (Scholl and Merril, 2005).

Bacteriophages (phages) are viruses that infect bacteria. Their potential to kill bacteria was first discovered in 1917 (D'Herelle, 1917), but despite their undaunted therapeutic potential, they were widely disregarded as therapeutic agents in Western nations due to the discovery of small-molecule antibiotics (Martha et al., 2011). However, the worldwide rise of multi-drug resistant bacteria has led to a reinvestigation of phage therapy as an alternative to antibiotics in human and animal infections. T7 is a small bacteriophage with a genome of ~40 kbp encapsulated in a 55-nm icosahedral head (Xu et al., 2018), which infects *E. coli* and related enteric bacteria, encoding a tail fibre that specifically binds to lipopolysaccharide and recognises many *E. coli* K12 strains (Scholl et al., 2005). Laboratory strains of K12 are similar to and share many genes and phenotypes with pathogenic strains, for example, *E. coli* O157:H7, so inhibitors of these strains are also likely effective against pathogens (Koli, 2021; Browning et al., 2013; Mahata et al., 2021). The K1F phage is a natural T7-like phage that infects *E. coli* O18:K1:H7. It is similar to T7 at the genome scale, although rather than the T7 tail fibre protein, it incorporates the endosialidase enzyme into its tail structure, allowing attachment to and degradation of the K1 polysaccharide capsule of its host (Scholl and Merril, 2005).

The bacterial filamentous temperature sensitive Z (FtsZ) protein is a tubulin homolog required for cell division in most species, including *E. coli (* Prahathees and Eswara, 2017). It is comprised of a globular domain, made up of two subdomains, which can fold independently (Moore et al., 2017). FtsZ monomers in the cytoplasm undergo GTP-dependent polymerisation into single-stranded protofilaments, which bundle together through lateral interactions (Haeusser et al., 2014). FtsZ polymerises to assemble a ring structure (called the Z-ring) at the division site, which recruits other cell division components, such as FtsI and ZapA (Szwedziak and Löwe, 2013), ready to initiate cytokinesis (Prahathees and Eswara, 2017). Since FtsZ is the first protein in the divisome and is needed for downstream recruitment, it would make a good target for novel antibiotics (Broughton et al., 2016; Araya et al., 2019), specifically aiming to inhibit FtsZ polymerisation (Dow et al., 2015). Changes in the polymerisation of FtsZ or GTPase activity would prevent the formation of the Z-ring and septum formation, which would in turn block cell division and cause cell death, making FtsZ a potential target for new antibacterial agents, as demonstrated by the mode of action of PC1900723 (Haydon et al., 2008). The Kil peptide of bacteriophage λ prevents FtsZ polymerisation and therefore Z-ring formation, leading to filamentation of *E. coli* cells and cell death (Haeusser et al., 2014). Kil has been shown to block FtsZ from forming protofilaments during the lytic cycle, and it has been suggested that Kil can directly interact with FtsZ *in vitro* for ZipA-dependent inhibition of the Z-ring (Haeusser et al., 2014). At high concentrations, Kil may be able to sequester FtsZ subunits and reduce their GTPase activity (Hernandez-Rocamora et al., 2015). The gene product (gp) 0.4 protein expressed by bacteriophage T7 prevents FtsZ assembly to give elongated bacterial cells, thereby enhancing T7’s competitiveness (Kiro et al., 2013). Gp0.4 is a non-essential gene transcribed approximately 2 min after infection with the early genes (Studier, 1972). It has previously been shown that purified FtsZ is inhibited by purified gp0.4 to block Z-ring assembly *in vitro*, along with *in vivo* studies, which found that gp0.4 specifically binds to and interacts with FtsZ (Kiro et al., 2013).

Several features of FtsZ make it an attractive drug target: it performs an essential set of functions in the majority of bacterial pathogens (Broughton et al., 2016); it has a specific role in prokaryotic cell division related to cell wall biosynthesis, which is a proven target for antimicrobial agents; it is conserved across the vast majority of bacterial and archaeal species (Prahathees and Eswara, 2017); it is absent in human and animals, so it should not exhibit adverse effects on host cells (Tripathy and Sahu, 2019).

The aim of this study was to investigate the effect of bacteriophages and their peptides on the localisation of FtsZ during infection. We used a previously developed *in vitro* meningitis model system (Moller-Olsen et al., 2020) to study the effect of bacteriophages on FtsZ using fluorescent *E. coli* EV36/FtsZ-mCherry (Vimr and Troy, 1985; Galli and Gerdes, 2010) and K12/FtsZ-mNeon (Moore et al., 2017) strains in the hCMEC human brain cell line. We investigate FtsZ localisation in both intracellular and extracellular bacteria within a human cell environment, in order to understand better the cell biology mechanisms during phage therapy. We show localisation of FtsZ to the bacterial cell midbody as a single ring during normal growth conditions in the absence and presence of human cells and mislocalisation of FtsZ to give filamentous multi-ringed cells upon addition of the known inhibitor Kil peptide (Haeusser et al., 2014). We show that bacteriophages K1F-GFP (Moller-Olsen et al., 2018) and T7-mCherry are able to inhibit FtsZ and block bacterial cell division in the exponential growth phase only since an actively dividing host is needed. We show that inhibition of the divisome proteins FtsI and ZapA occurs, and potentially the inhibition of MinC. Finally, we show that the T7 peptide gp0.4 is responsible for inhibition of FtsZ in K12 strains by observing a phenotype recovery with a T7Δ0.4 mutant (Kiro et al., 2013), and this peptide potentially acts to delay host cell lysis and increase phage progeny numbers.

## 2 Materials and methods

### 2.1 Human cell culture

The human cerebral microvascular endothelial cell (hCMEC) line (Merck, London, UK) was cultured in EndoGRO-MV Complete Media (Merck) supplemented with 1% penicillin–streptomycin and grown at 37°C with 5% CO_2_ in culture vessels coated with 5 µg/cm^2^ Collagen Type 1 (Merck). Prior to the experiment, the cells were seeded onto coverslips in a 6-well plate or a fluorodisc in culture media at a density of 4 × 10^4^ cells/ml and left for 24 h to settle. The culture media was then replaced with Leibovitz L-15 media (Lonza), and the cells were moved to a 37°C incubator suitable for bacterial infection.

### 2.2 Bacterial cultures

Bacterial cultures were grown from a single colony in lysogeny broth (LB), supplemented with the appropriate antibiotic if needed, and grown in a 37°C shaking incubator. The seven bacterial strains used in this study are listed in **Table 1**. During experiments, bacterial cells were added during the exponential growth phase at OD_600_ 0.3 (unless otherwise stated as stationary) and incubated for 1 h prior to fixation or phage addition.

**Table 1:** Bacterial strains used in this study.

Strain	Description	Source
*Escherichia coli* EV36	*E. coli* K1/K12 hybrid suitable for Class 1 laboratory work while maintaining phenotypic properties of a pathogenic K1 strain	Dr Eric R. Virm (Vimr and Troy, 1985)
*E. coli* EV36/FtsZ-mCherry	Derivative of EV36 transformed with a pBAD30FtsZ-mCherry plasmid and cultured under 35 µg/ml chloramphenicol selection	Plasmid kindly provided by Dr Kenn Gerdes (Galli and Gerdes, 2010), transformed in this study
*E. coli* EV36/Kil	Derivative of EV36 transformed with a pBAD33-Kil plasmid and cultured under 35 µg/ml chloramphenicol selection	Plasmid kindly provided by Dr William Margolin (Haeusser et al., 2014), transformed in this study
*E. coli* K12 FtsZ-mNeon	*E. coli* BW27783 strain, which has had its wild-type *ftsZ* gene replaced with FtsZ-mNeonGreen	Dr Harold Erickson (Moore et al., 2017)
*E. coli* K12/FtsZ-mNeon/Kil	Derivative of K12/FtsZ-mNeon transformed with a pBAD33-Kil peptide plasmid and cultured under 35 µg/ml chloramphenicol selection	Plasmid kindly provided by Dr William Margolin (Haeusser et al., 2014), transformed in this study
*E. coli* K12ΔzapA	*E. coli* BW25113 K12 strain from the Keio collection with a deleted *zapA* gene that has been replaced by a kanamycin resistance cassette. Cultured under 50 µg/ml kanamycin selection	OEC4987-200828117, Keio collection (Baba et al., 2006)
*E. coli* K12ΔminC	*E. coli* BW25113 K12 strain from the Keio collection with a deleted *minC* gene that has been replaced by a kanamycin resistance cassette. Cultured under 50 µg/ml kanamycin selection	OEC4987-213605848, Keio collection (Baba et al., 2006)

### 2.3 Bacteriophage propagation and purification

The bacteriophages used in this study are listed in **Table 2**. *E. coli* EV36 was used as a host to grow and purify K1F-GFP phage, and K12/FtsZ-mNeon was used as a host to grow and purify T7 phage, T7-mCherry phage, and T7Δ0.4 phage. After host clearance, each phage propagation culture was centrifuged at 3,220 × *g* for 15 min. The resulting supernatant was filter sterilised and incubated on ice with 0.2 M of NaCl for 1 h and then centrifuged at 3,220 × *g* for 15 min at 4°C. The supernatant was then incubated with 10% w/v PEG8000 overnight at 4°C to precipitate the phage and then centrifuged at 3,220 × *g* for 15 min at 4°C. The resulting phage pellet was resuspended in an SM buffer 1 (1 M of NaCl, 8 mM of MgSO_4_•7H_2_O, and 25 mM of Tris-HCl). A CsCl density gradient was set up with three solutions of densities of 1.7, 1.5, and 1.4 g/ml, along with a phage solution with CsCl added to give a density of 1.3 g/ml. The solutions were added in equal volumes to a centrifuge tube, starting with the heaviest, and centrifuged at 125,000 × *g* for 20 h at 4°C. The resulting phage band was extracted and placed in dialysis tubing (molecular weight cutoff (MWCO) of 14 kDa) and dialysed overnight in SM buffer 1 at 4°C, followed by dialysis in SM buffer 2 (100 mM of NaCl, 8 mM of MgSO_4_•7H_2_O, and 25 mM of Tris-HCl) for 2 h at room temperature twice. After dialysis, the purified phage was retrieved and stored at −20°C, and the phage titre was found by plaque assay. WT T7, T7-mCherry, and K1F-GFP phage stocks were diluted to approximately 10^9^ pfu/ml for use in experiments. For the experiments, phages were incubated for 1 h prior to fixation.

**Table 2:** Phage strains used in this study.

Phage	Description	Source
K1F-GFP	A K1F phage derivative engineered to express GPF, which shows a high specificity towards K1 capsule bacteria	K1F from Dr Dean Scholl (Scholl and Merril, 2005), K1F-GFP from Dr Antonia Sagona (Moller-Olsen et al., 2018)
T7	Extensively used strain showing high specificity to commensal *E. coli* K12 strains	Dr Ian Molineux, Texas USA (Studier, 1969)
T7-mCherry	A T7 phage derivative engineered with genomic integration of mCherry, showing high specificity to commensal *E. coli* K12 strains	This study
T7Δ0.4	T7 phage derivative engineered with a knockout of gp0.4 showing high specificity towards commensal *E. coli* K12	Dr Udi Qimron (Kiro et al., 2013)

### 2.4 Engineering of T7-mCherry phage

#### 2.4.1 Plasmid construction

The pSB1C3 plasmid (Registry of Standard Biological Parts http://parts.igem.org/Main_Page) contained chloramphenicol resistance. The synthetic gBlock was ordered from Integrated DNA Technologies, with *Eco*RI and *Spe*I sites to ligate to the corresponding restriction sites of the pSB1C3 plasmid. The gBlock (**Figure S1**) was designed to contain the mCherry gene (Shaner et al., 2004) flanked by 150-bp homology regions of the C-terminus (excluding the stop codon) of the T7 minor capsid protein (gene 10), along with a linker sequence. A PCR was performed to amplify the insert using primers AG005 and AG006 (**Table S1**). The insert and vector were digested with restriction enzymes at 37°C for 1 h, purified using a GeneJET PCR Purification Kit (Thermo Fisher Scientific, Waltham, MA, USA), and then ligated (150 ng of insert and 50 ng of vector) with DNA T4 ligase overnight at 4°C.

#### 2.4.2 Sequencing the plasmid

After ligation, the resulting vector was transformed into electrocompetent K12 cells and plated on chloramphenicol agar overnight, and plasmid was miniprepped from the colonies using a QIAprep Spin kit. The miniprepped DNA was sent for Sanger sequencing by GATC using the AG005 primer. One plasmid returned a matching sequence and was used for engineering the phage.

#### 2.4.3 Homologous recombination

Wild-type T7 phages were propagated with electrocompetent K12/FtsZmNeon transformed with the donor plasmid at a low OD_600_ of 0.2. Homologous recombination occurred between the homology regions of the plasmid and genome of some phages, resulting in a mixed population lysate, which was centrifuged at 3,220 × *g* for 15 min and then filter sterilised.

#### 2.4.4 Screening for engineered phage

The presence of engineered phage was found by PCR analysis of the lysate using the primers mCherry-forward and mCherry-rev (**Table S1**). The positive lysate was then plated out on the host lawn at an appropriate dilution to give individual phage plaques. Ten plaques were chosen and used as a template for a second PCR screen. The plaques showing positive bands were propagated as before, the lysate was plated for a second plaque assay, and plaques were again checked by a PCR screen. Positive phage was propagated, CsCl purified, and further checked for mCherry fluorescence by confocal and electron microscopy.

### 2.5 Bacterial and phage infections of human cells

Cultures of hCMECs were incubated with the relevant bacterial strain at OD_600_ of 0.2–0.4, which were added to the Leibovitz media for 60 min (unless otherwise stated) along with antibiotics or plasmid induction if needed, and if phages were being used, then the phage was added at 1 × 10^7^ pfu/ml and incubated for a further 60 min. Control cultures were incubated with bacteria or phage alone for 60 min in parallel.

To investigate if strains acted intracellularly, hCMEC cultures were infected with bacterial cultures at OD_600_ 0.3 for 1 h and then incubated with 100 μg/ml of gentamicin for a further 2 h to kill extracellular bacteria, performed in triplicate for each condition. For each condition, a minimum of 300 human cells were counted to calculate the internalised bacteria percentages.

To quantify human cell death after bacterial infection, hCMEC cultures were infected with bacterial cultures at OD_600_ 0.3 for 1 h, in triplicate conditions. For each condition, a minimum of 500 human cells were counted.

### 2.6 Immunofluorescent confocal microscopy

After bacterial and/or phage infection, hCMEC cultures were fixed with 4% paraformaldehyde (Thermo Fisher Scientific) in phosphate-buffered saline (PBS) for 15 min and then washed in PBS. Cells were then permeabilised in ice-cold PEM/0.05% saponin for 5 min, washed, incubated with 50 mM of NH_4_Cl in PBS for 15 min, and then washed in PBS/0.05% saponin. The fixed cells were stained overnight at 4°C with anti-Prokaryotic Cell Division GTPase (FtsZ) antibody (Agrisera, Vännäs, Sweden) or rabbit anti-*E. coli* (strain K12) FtsI Polyclonal antibody if no fluorescent proteins were present and FtsZ or FtsI staining was needed, with diluted 1:100 in 0.05% saponin in PBS. This was followed by conjugation with secondary Donkey Anti-Rabbit IgG H&L (Alexa Fluor^®^ 488) diluted 1:500 in 0.05% saponin in PBS at room temperature for 45 min. For samples with fluorescent proteins, the fixed cells were stained with the following antibodies diluted in PBS/0.05% saponin for 60 min at room temperature: 5 µg/ml of GFP-booster (ChromoTek, Planegg, Germany), 5 µg/ml of RFP-booster (ChromoTek), and 5 µg/ml of phalloidin CF680R conjugate (Biotium, Fremont, CA, USA).

After staining, the coverslips were washed, mounted on slides with DAPI-containing Fluoroshield Mounting Medium (Abcam, Cambridge, UK), and sealed. The slides were then imaged using a Zeiss LSM800 confocal microscope using the following excitation wavelengths: DAPI at 405 nm, GFP/mNeon at 488 nm, mCherry at 561 nm, and phalloidin at 633 nm.

Quantification was performed by manually counting Z-rings of 75 bacterial cells per condition and measuring cell length using the Fiji (ImageJ) software for phage filamentation experiments. Data were plotted with error bars showing one standard deviation of uncertainty. For significant difference comparisons, Tukey’s tests were performed, and the calculated probability values (p-values) are displayed as p < 0.05 (*), p < 0.01 (**), and not statistically significant p > 0.05 (ns).

### 2.7 Live-cell imaging

Cultures of bacteria alone expressing fluorescent proteins were imaged live on 2% agarose pads. Cultures of K12/FtsZ-mNeon were grown to OD_600_ 0.5 and imaged immediately. For EV36/FtsZ-mCherry, cultures were grown to OD_600_ 0.3 and induced with 0.2% arabinose for 5 min (stopped using 0.2% glucose) and incubated for 45 min to allow time for protein folding before imaging. Agarose pads were made by dissolving 0.2 g of agar into 10 ml of sterile water by heating, and a drop was placed onto a glass microscope slide and left to dry. Culture measuring 10 µl was then placed onto the agarose layer and covered with a coverslip once dry. Cells were imaged using the Zeiss LSM 880 confocal microscope, with mNeon excitation at 488 nm and mCherry at 561-nm wavelengths, and transmitted light was used to visualise the cell outline.

### 2.8 Correlative light and electron microscopy

T7-mCherry phage was added to a K12/FtsZ-mNeon culture (OD_600_ 0.3) and incubated at 37°C with shaking for 60 min; 300 mesh copper grids (FC300Cu) were hydrophilised by glow discharge. The sample was added to the grid and incubated for 2 min, and then 2% uranyl acetate was dropped into the grid and incubated for 2 min. A wash step was performed three times and blotted; 0.1% trehalose was added to the grid and incubated for 2 min. This was left to air-dry. The sample was imaged using Zeiss LSM 880 confocal microscope (565 nm for T7-mCherry and 488 nm for K12/FtsZ-mNeon) and Jeol 2100 transmission electron microscope, and ImageJ was used for overlay.

## 3 Results

### 3.1 Visualisation of the FtsZ ring and Kil peptide-mediated inhibition of FtsZ

We first sought to observe Z-rings in bacterial cells, using the *E. coli* K12/FtsZ-mNeon (Moore et al., 2017) strain, which has its genomic copy of *FtsZ* gene replaced with FtsZ-mNeon. A crucial aspect of the design of this FtsZ construct is that mNeon protein has been placed on an internal loop of FtsZ so that the head-to-tail mode of polymerisation required for the function was not impaired (Moore et al., 2017), and the mNeon proteins were prevented from forming aggregates with each other. The Z-rings were visualised by placing exponential-phase bacterial cultures onto 2% agarose pads for visualisation by confocal microscopy. FtsZ-mNeon assembled at the cell midbody as a single Z-ring (**Figure S2A**). This visualisation was repeated in the *E. coli* K1/K12 hybrid strain EV36 (Vimr and Troy, 1985), by transforming it with an arabinose-inducible FtsZ-mCherry plasmid (Galli and Gerdes, 2010), and FtsZ-mCherry forms a Z-ring at the EV36 midbody (**Figure S2B**).

**Figure 1:**
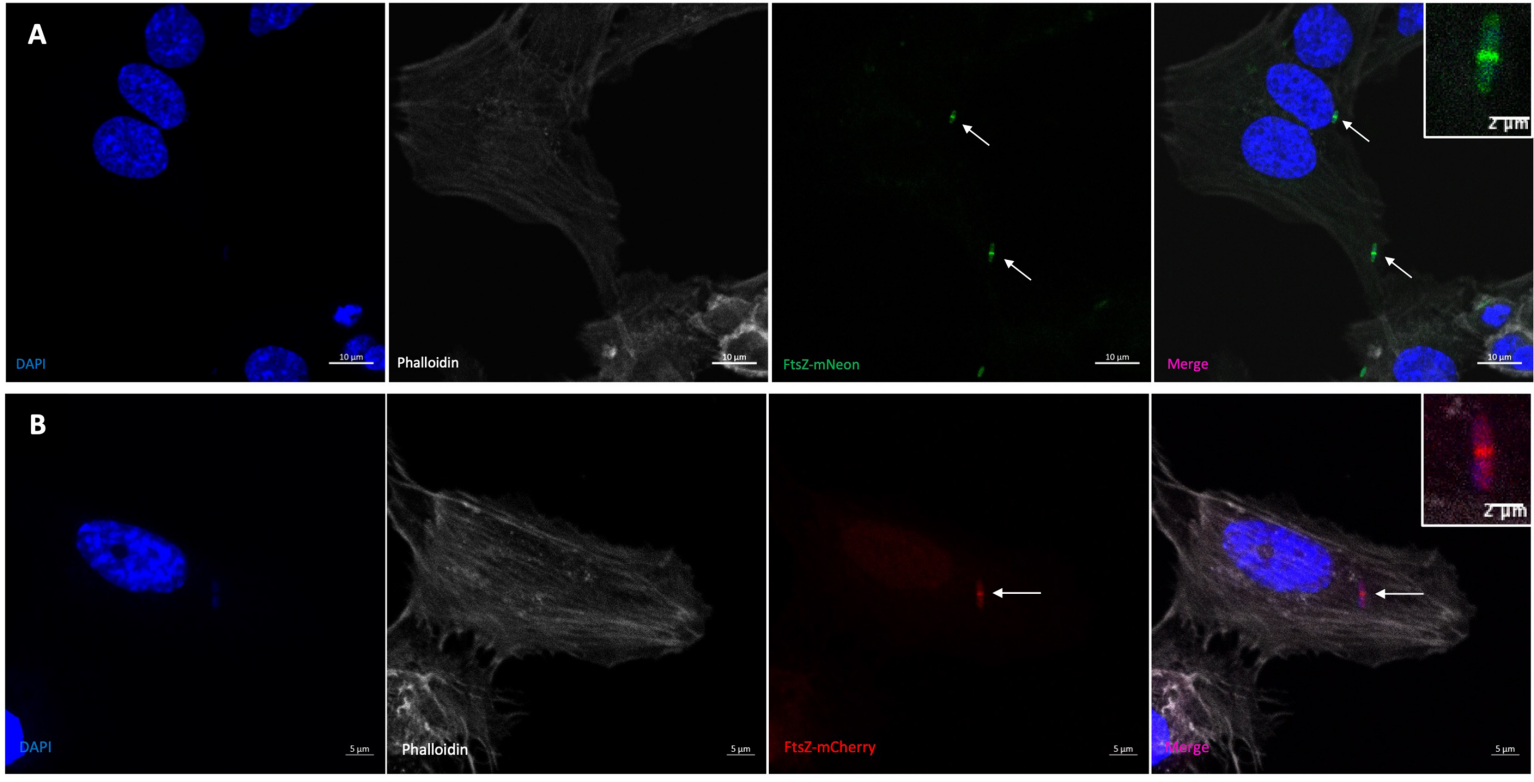
Visualisation of the FtsZ Z-ring in normal growth conditions by confocal microscopy. **(A)** K12/FtsZ-mNeon cells fixed during human cell infection. An internalisation experiment using gentamicin to kill extracellular bacteria showed this strain had an infection rate of 4.1% in hCMECs. **(B)** EV36/FtsZ-mCherry cells fixed during human cell infection. Arrows pointing to bacterial Z-rings. DAPI stain is shown in blue, phalloidin stain is shown in grey, FtsZ-mNeon is shown in green, and FtsZ-mCherry is shown in red.

We next observed the phenotype of the Z-rings in a meningitis infection model to see if FtsZ localisation changed during bacterial infection of human cells. hCMEC brain cells were infected with K12/FtsZ-mNeon culture for 1 h, fixed, and stained before visualising by confocal microscopy. During human cell infection, FtsZ rings were still visible at the midbody of K12 cells as a single ring per cell (**Figure 1A**), suggesting cells can divide normally, and this was also found for the EV36/FtsZ-mCherry strain (**Figure 1B**). These data confirm that FtsZ assembles as a single ring at the cell midbody under normal bacterial growth conditions and during bacterial infection of human cells. The EV36 strain has previously been shown to have an infection rate of 20%–50%, depending on the human cell line (Moller-Olsen et al., 2018; Moller-Olsen et al., 2020). Since the K12 strain is non-pathogenic and not expected to act intracellularly (Meier et al., 1996; Sahu and Kar, 2012), an internalisation experiment was done using gentamicin to kill extracellular bacteria and to confirm if K12 would be present inside human cells. This showed that only 4.1% of hCMECs infected with K12/FtsZ-mNeon had intracellular bacteria. Since the infection rate is low, for the following experiments, we chose to focus on both intracellular and extracellular bacteria in the infection model for both strains. Nevertheless, both extracellular and intracellular bacteria are toxic to human cells and have a variety of mechanisms to cause disease (Mak et al., 2014). We confirm this by quantifying human cell death by confocal microscopy after bacterial infection of human cells and found that 5.3% of human cells died after infection with the K12 strain, and 12.1% of human cells died after infection with the EV36 strain (**Figure S3**). Recent studies have presented the efficiency of bacteriophages in targeting both intracellular and extracellular bacteria in the human cell environment (Śliwka et al., 2022), and bacteriophages have been shown to be more efficient in the presence of human cells (Shan et al., 2018). It is therefore necessary to test the proposed system in infection human cell models, in order to understand better the interplay of phage and bacteria in the presence of human cells.

We then applied the known FtsZ inhibitor Kil peptide to the model system to investigate how FtsZ inhibition would appear in these conditions. Kil stops FtsZ from polymerising, leading to filamentation of cells that are unable to divide, so a Kil peptide plasmid (Haeusser et al., 2014) was transformed into both strains. For these experiments, the plasmid was induced with 0.2% arabinose for 2 h. Imaging in **Figure S4A** confirmed that induction of Kil in K12/FtsZ-mNeon resulted in long filamentous cells, with varying FtsZ phenotypes that ranged from distinct multiple rings (42.2% of cells) to diffuse FtsZ spread (30.1%) and cells in an intermediate phenotype (27.7%) of faint rings within a diffuse background. Previous studies have shown a diffuse FtsZ spread upon Kil inhibition using antibody staining (Haeusser et al., 2014), which was confirmed in **Figure S5**; however, here we present that when using a fluorescent protein stably expressed, multiple distinct Z-rings still form along the cell body, potentially due to Kil not having fully acted on these cells yet.

To support these findings, images for the induction of Kil were quantified in K12/FtsZ-mNeon under the following conditions: control (no Kil induction), 1-h induction, and 2-h induction. The averages (**Figures S4C** **, D**) showed an increase in the number of Z-rings and cell length after 1 h of Kil induction, with a further increase after 2 h, confirming that the cells are not dividing and continually elongating in the presence of Kil, and formation of multiple Z-rings suggests cell division had been unsuccessfully attempted. The increase seen after 2 h compared to 1 h suggests that the effect and phenotypes seen may be dependent on the amount of Kil peptide present in the cell.

These data support previous findings that the phage λ Kil peptide causes filamentation of *E. coli* cells by blocking cell division with diffuse FtsZ spread by antibody staining, as FtsZ is unable to polymerise to form rings and further show that multiple Z-rings can form along the body of the filamentous cell, as it fails to divide at new division sites when using fluorescent proteins.

### 3.2 Genetic engineering of a T7-mCherry phage

**Figure 2:**
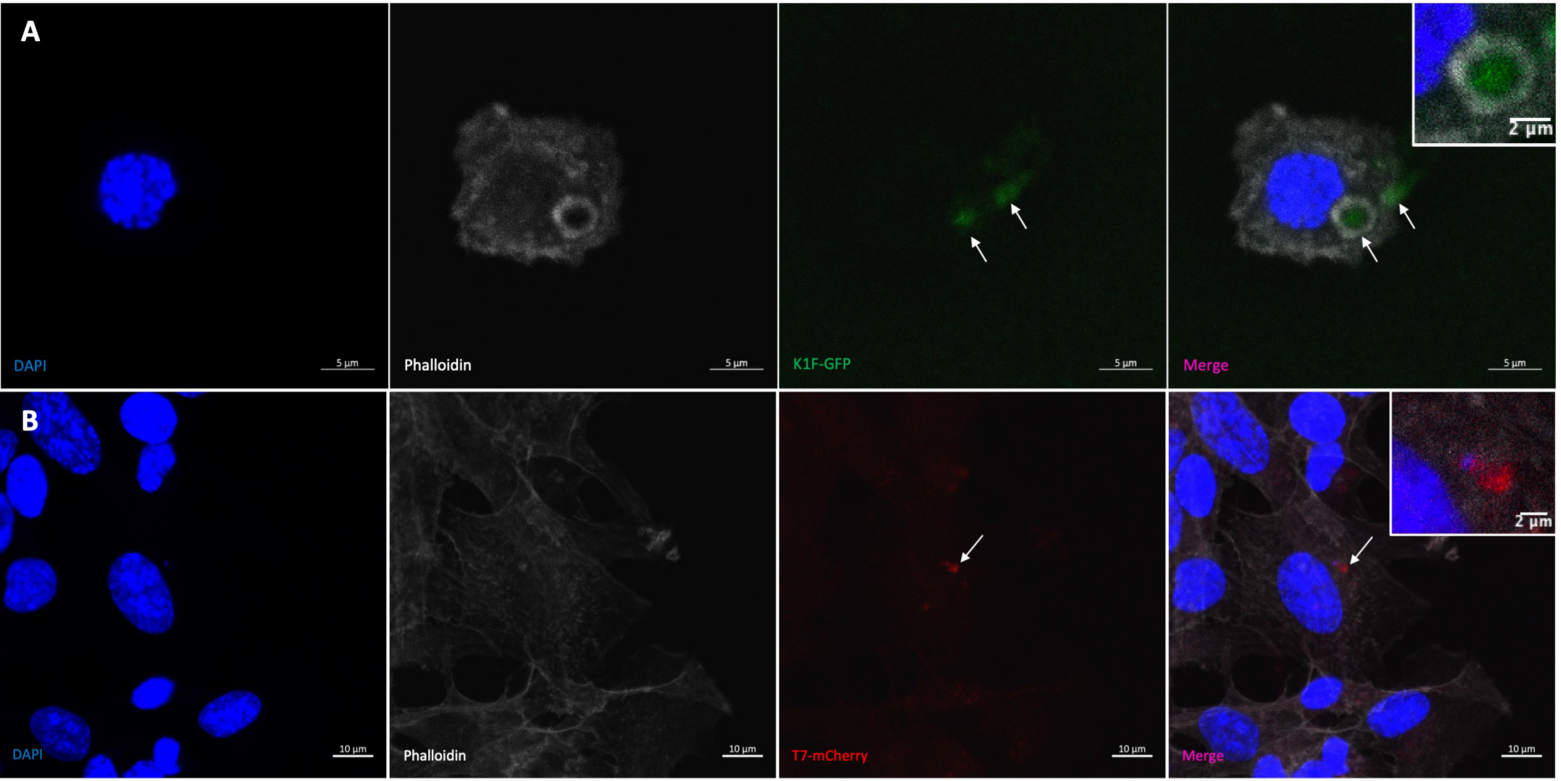
Visualising bacteriophage in hCMEC brain cells. **(A)** K1F-GFP phage inside vacuole of hCMEC cell after infection. **(B)** T7-mCherry phage inside hCMECs after infection. Arrows pointing to phage clusters. DAPI stain is shown in blue, phalloidin stain is shown in grey, K1F-GFP is shown in green, and T7-mCherry is shown in red.

The K1F-GFP phage (Moller-Olsen et al., 2018) has been previously engineered to express GFP, and this was first visualised in human cell vacuoles (**Figure 2A**) to confirm fluorescence compared to no-phage controls as a visual control for use in confocal experiments. In order to visualise the T7 phage in the K12/FtsZ-mNeon model system, we attached a fluorescent protein to the C-terminal of the phage’s minor capsid protein so that it is displayed at the surface, using homologous recombination from an engineered plasmid. The presence of mCherry gene in the phage lysate was confirmed by PCR screening of the recombinant phage genome using the primers in **Table S1** and associated plaque assays (**Figures S6A** **–D**), prior to purification of positive lysate by caesium chloride gradient purification (**Figures S6E** **, F**). Fluorescence of the mCherry tag was further tested by confocal microscopy (**Figure 2B**) showing mCherry fluorescence inside human cells after phage infection, alongside no-phage controls (**Figure S7**), performed to show the fluorescence seen was from the phage label, confirming successful engineering of the T7-mCherry phagemid, which was able to be taken up by human cells.

### 3.3 T7 phage blocks cell division in K12 by FtsZ inhibition

K1F-GFP phage was confirmed to target the EV36/FtsZ-mCherry strain (**Figure S8A**), and T7-mCherry phage was confirmed to target the K12/FtsZ-mNeon strain (**Figure S8B**) by using growth curves. This was further confirmed by correlative light and electron microscopy (CLEM) showing the formation of a septum at the midbody of cells, as well as colocalisation of FtsZ-mNeon and T7-mCherry signals within K12 cells (**Figure S9**), confirming the specificity of the phage.

**Figure 3:**
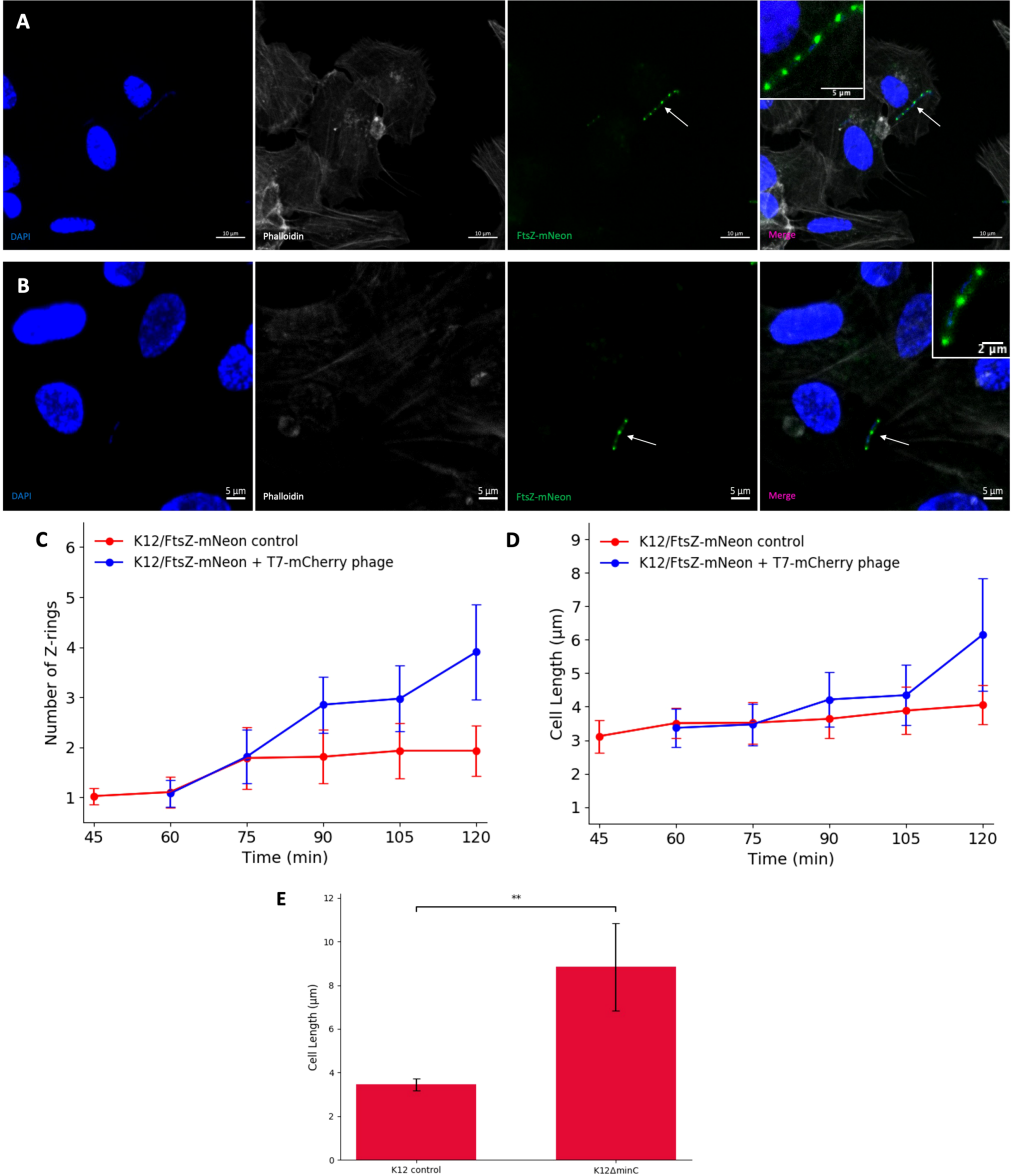
T7 phage inhibition of FtsZ in K12 *Escherichia coli* cells. **(A, B)** Fluorescent images of K12/FtsZ-mNeon cells after T7-mCherry phage infection fixed in a human cell model. Arrows point to filamentous cells. DAPI stain is shown in blue, phalloidin in grey, and FtsZ-mNeon in green. **(C, D)** Quantification of Z-ring number and cell length for 75 K12/FtsZ-mNeon bacterial cells infected with T7-mCherry phage at 15-min intervals and compared to a no-phage control. Averages plotted with error bars showing one standard deviation of uncertainty. **(E)** Quantification of cell length for 75 K12 control and K12ΔminC bacterial cells. Tukey’s tests were performed, and the calculated probability values (p-values) are displayed as p < 0.01 (**).

Next, the effect of T7-mCherry on FtsZ was examined by infecting hCMEC with K12/FtsZ-mNeon, followed by incubation with T7-mCherry. Confocal microscopy showed that T7 phage infection caused a change from the single midbody ring previously seen and led to the formation of filamentous, multi Z-ring *E. coli* cells (**Figures 3A** **, B**), which were unable to divide, suggesting that T7 phage can block cell division. We were then interested to see how the Z-rings and cell length of *E. coli* changed over time in control conditions (no phage) and the presence of T7 phage by using a time-series experiment. For the control, hCMECs were infected with exponential K12/FtsZ-mNeon and fixed at 15-min intervals for 2 h (**Figure S10**). For the phage time series, hCMECs were infected with K12/FtsZ-mNeon for 1 h, and then T7 phage was added (**Figure S11**). For each condition, bacterial cells were quantified using confocal microscopy (**Figures 3C** **, D**). Since K12/FtsZ-mNeon cells were incubated prior to phage addition, this meant phage time of 0 min was equivalent to control 60 min, so these were plotted to overlap. Larger error bars were due to the variation in phenotypes, as some cells were fully filamentous, whereas others were yet to be infected. In the first 60 min of the control series, each cell averages one Z-ring at the midbody as seen earlier in **Figure 1**, but later we unexpectedly observed two 2 polar rings in the majority of cells. Upon phage addition, the number of Z-rings started to increase after 15–30 min, and after 1 h, they reached an average of four rings, double compared to the control. After 1 h of incubation with phage, there was a notable increase in cell length showing filamentation, but interestingly, this did not start until about 30–45 min after infection, suggesting that the number of rings started to increase first and then filamentation followed.

To investigate the cause of the filamentation, confocal microscopy was performed using a K12ΔminC strain from the Keio collection with *minC* gene knocked out, alongside a K12 control. Cultures were incubated for 2 h in no-phage conditions, then stained with an FtsZ antibody, and imaged (**Figure S12**). The images were quantified, and average cell length was plotted (**Figure 3E**), showing a significant increase in cell length of K12ΔminC compared to the control, suggesting that inhibition of MinC could be causing the filamentation seen upon phage infection of cells.

Taken together, these data confirm the specificity of engineered T7-mCherry for K12 strains and show that during infection, the phage causes a filamentous (potentially due to MinC inhibition) non-dividing phenotype with multiple Z-rings formed where the division had been attempted, similar to what was seen with the known FtsZ inhibitor Kil, suggesting that T7 phage can inhibit FtsZ to block division.

### 3.4 K1F phage blocks cell division in EV36 K1 by FtsZ inhibition

**Figure 4:**
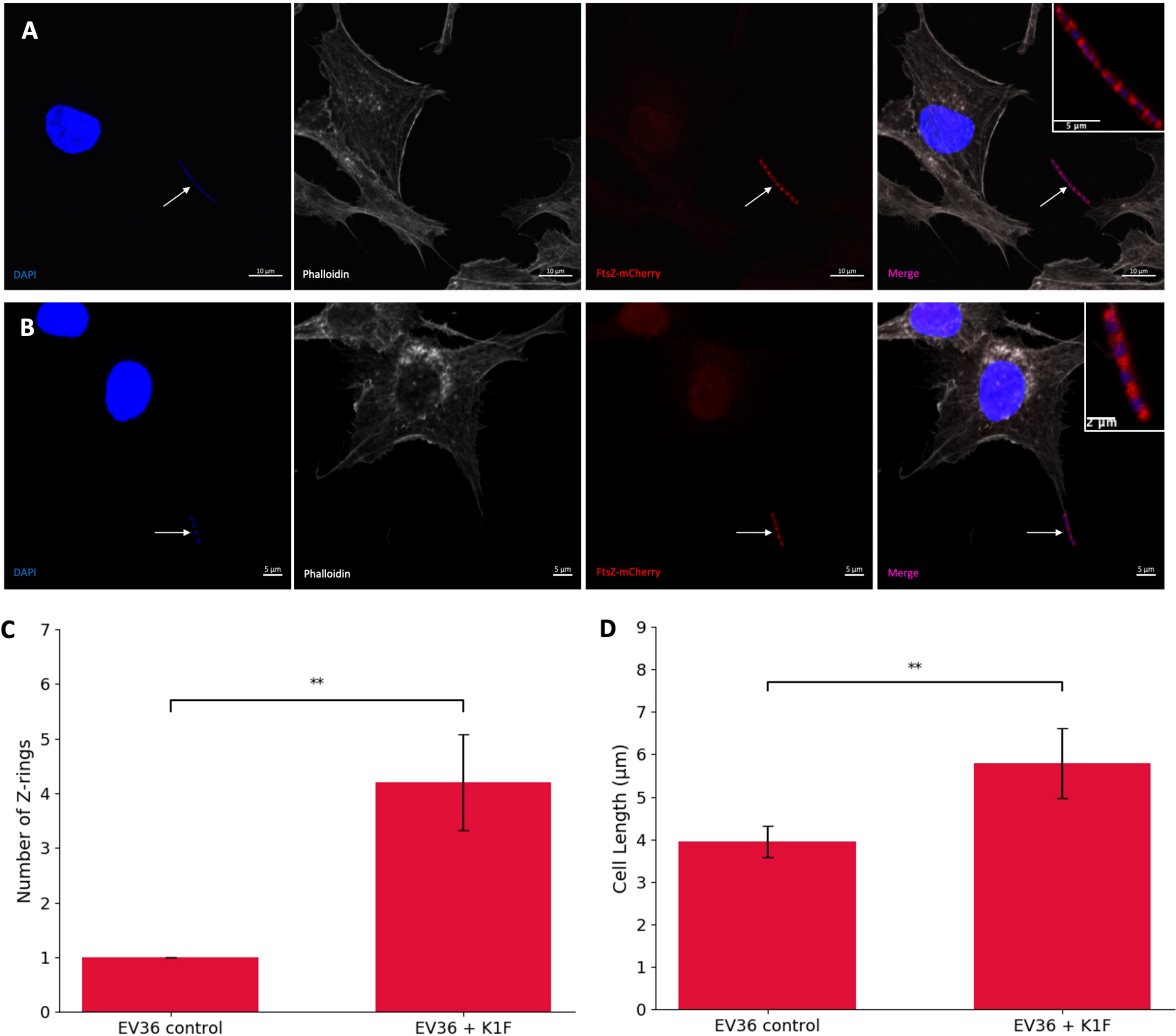
K1F phage inhibition of FtsZ in K1 *Escherichia coli*. **(A, B)** Fluorescent images of EV36/FtsZ-mCherry cells after K1F-GFP phage infection fixed in a human cell model. Arrows point to filamentous cells. DAPI stain is shown in blue, phalloidin in grey, and FtsZ-mCherry in red. **(C, D)** Quantification of Z-ring number and cell length for 75 EV36/FtsZ-mCherry bacterial cells infected with K1F-GFP phage and compared to a no-phage control. Averages plotted with error bars showing one standard deviation of uncertainty. Tukey’s tests were performed, and the calculated probability values (p-values) are displayed as p < 0.01 (**).

To investigate the effect of K1F-GFP phage on FtsZ localisation, hCMECs were infected with host EV36/FtsZ-mCherry and K1F-GFP phage. The EV36/FtsZ-mCherry cells showed a long filamentous phenotype with multiple Z-rings and DNA along the cell after K1F-GFP infection (**Figures 4A** **, B**), unlike control samples, suggesting that the K1F phage can also inhibit FtsZ to block host cell division.

To further confirm this, bacterial cells were quantified for control conditions and K1F-infected conditions (**Figures 4C, D**). The control showed an average of 1.027 rings per cell as expected, and after K1F-GFP infection, this increases to 4.120 rings per cell, suggesting cells had attempted to divide two to three times unsuccessfully. There was an increase in average cell length, showing cells were starting to elongate to confirm filamentation. These data show that similar to phages λ and T7, the K1F phage is able to inhibit FtsZ to block host cell division.

### 3.5 Effects of T7 bacteriophage on ZapA and FtsI divisome proteins

**Figure 5:**
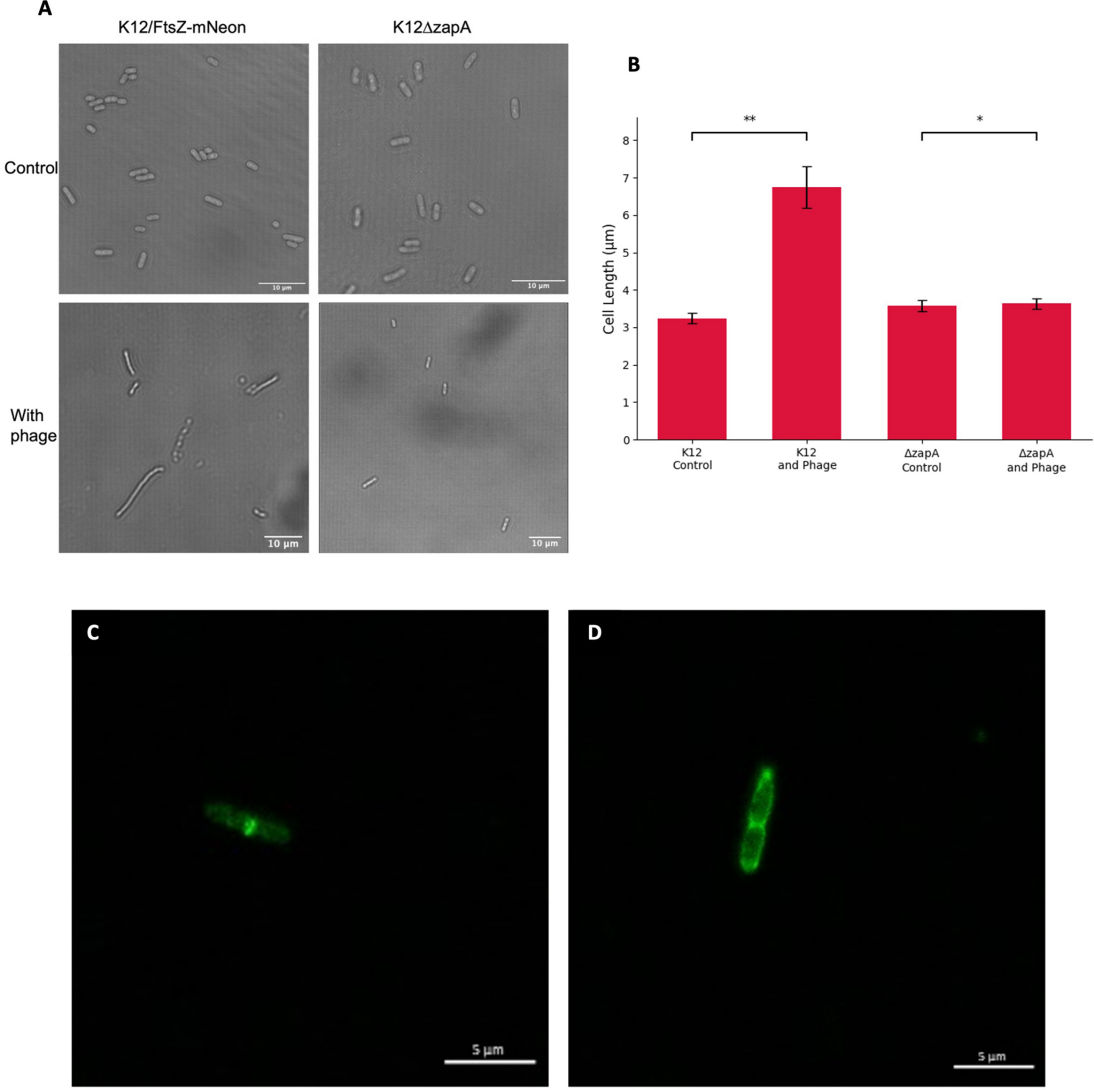
Effects of T7 bacteriophage on ZapA and FtsI divisome proteins. **(A)** Light microscopy images of K12 control and K12ΔzapA cells after T7 phage infection. **(B)** Quantification of cell length for 75 K12 control and K12ΔzapA bacterial cells infected with T7 phage and compared to a no-phage control. Averages plotted with error bars showing one standard deviation of uncertainty. Tukey’s tests were performed and the calculated probability values (p-values) are displayed as p < 0.05 (*), p < 0.01 (**), and not statistically significant p > 0.05 (ns). **(C)** *Escherichia coli* K12 cell stained with an FtsI antibody showing localisation of FtsI to the cell midbody as a single ring. **(D)** *E. coli* K12 cell after T7 phage infection stained with an FtsI antibody showing mislocalisation of FtsI to three rings.

We hypothesised that the observed effect of FtsZ inhibition by phages may not be specific to just FtsZ and that other divisome proteins may be involved in the infection process. To test this, a K12ΔzapA strain from the Keio collection with *zapA* gene knocked out was used, alongside a K12 control. Cultures were incubated for 1 h, then infected with T7 phage for a further hour, alongside a no-phage control, and imaged by light microscopy to observe cell filamentation (**Figure 5A**). The images were quantified, and average cell lengths for the conditions were plotted (**Figure 5B**). As previously shown, there was an increase in cell length upon T7 phage infection of K12 cells as they became filamentous, but this phenotype appears to be absent during T7 phage infection of K12ΔzapA cells, suggesting that ZapA is essential for cell filamentation and the phenotype seen during phage inhibition of cell division.

Next, the localisation of FtsI protein in this system was investigated. Cultures of K12 were grown for 1 h, infected with T7 phage, alongside a no-phage control, and stained with an FtsI antibody. Under normal growth conditions in the no-phage control, we observed that FtsI is localising to the midbody of the cell as a single ring (**Figure 5C**) as expected after recruitment by FtsZ as the divisome forms. After T7 phage infection of K12, we observed mislocalisation of FtsI (**Figure 5D**). This suggests that other divisome proteins are mislocalised upon phage infection as cell division is inhibited, potentially being recruited by mislocalised FtsZ.

#### 3.5.1 T7 Gp0.4 targets FtsZ to increase titres and delays lysis

**Figure 6:**
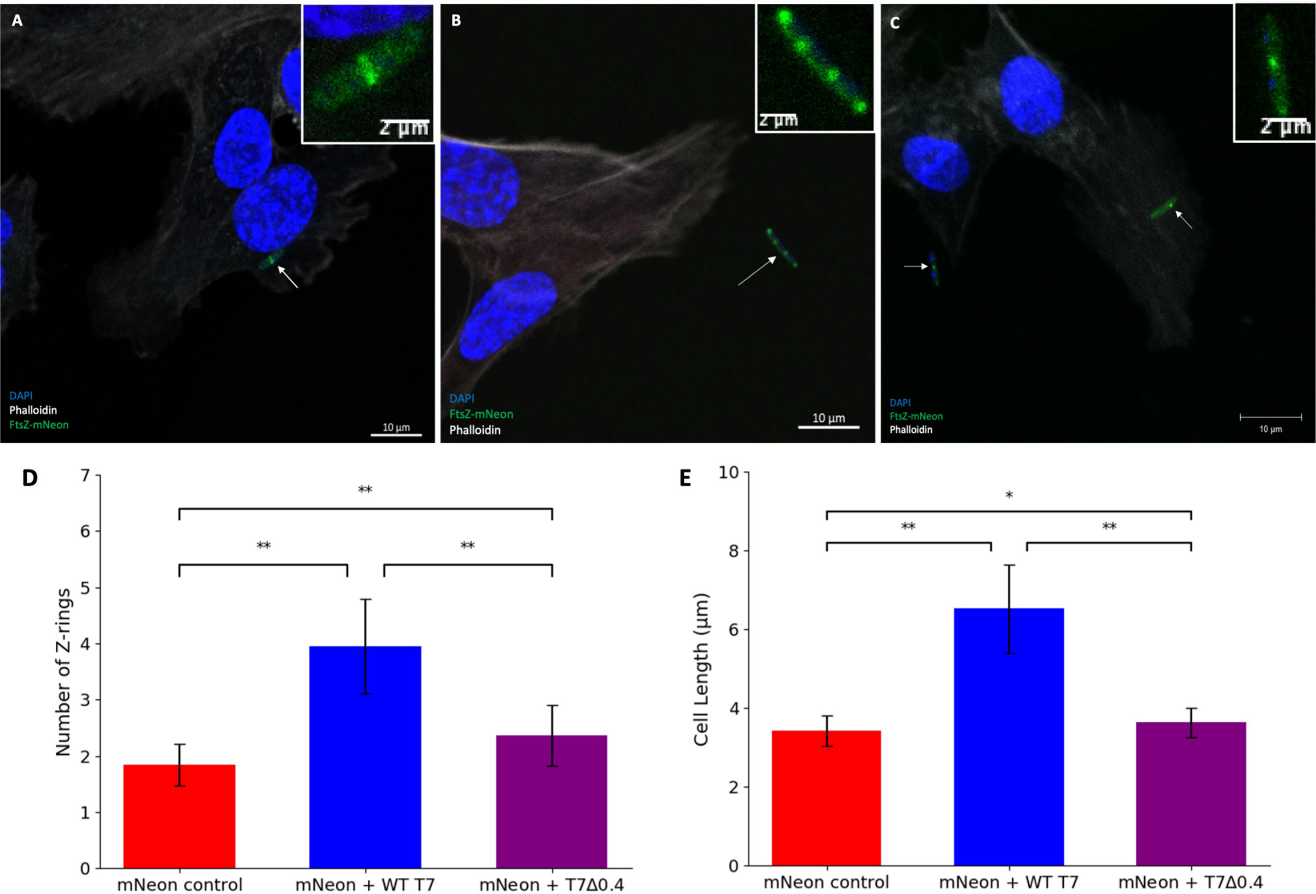
T7Δ0.4 mutant phage microscopy. **(A)** K12/FtsZ-mNeon control cells fixed in human cells. **(B)** K12/FtsZ-mNeon cells infected with T7-mCherry phage fixed in human cells. **(C)** K12/FtsZ-mNeon cells infected with T7Δ0.4 phage fixed in human cells. For fluorescent images, DAPI stain is shown in blue, phalloidin in grey, and FtsZ-mNeon in green. Arrows pointing to bacterial cells of interest. **(D,E)** Quantification of Z-ring number and cell length for 75 K12/FtsZ-mNeon bacterial cells infected with T7-mCherry or T7Δ0.4 phage during exponential growth and compared to a no-phage control. Averages plotted with error bars showing one standard deviation of uncertainty. Tukey’s tests were performed, and the calculated probability values (p-values) are displayed as p < 0.05 (*), p < 0.01 (**), and not statistically significant p > 0.05 (ns).

It has been previously shown by light microscopy that the T7 phage peptide gp0.4 directly inhibits FtsZ and causes host cell filamentation (Kiro et al., 2013), and in this study, we confirm this observation by confocal microscopy, to allow visualisation of the Z-rings in the human cell model after infection with a T7Δ0.4 mutant phage (Kiro et al., 2013). hCMECs were treated with K12/FtsZ-mNeon followed by infection with T7-mCherry or T7Δ0.4 phage, along with a control. Cells were imaged to see the resulting phenotypes (**Figures 6A** **–C**), and for each condition, cells were quantified and averages were plotted (**Figures 6D** **, E**). Upon T7Δ0.4 addition, unlike the WT T7, the mutant-infected *E. coli* showed recovery back to a phenotype similar to the control, confirming that T7’s gp0.4 targets FtsZ and causes filamentation of the cells as well as multiple Z-rings along the cell body. After 2 h, the control sample had an average of 1.920 rings per cell with an average length of 3.426 µm, and for the WT T7-infected cells, this nearly doubled to an average of 3.827 rings and 6.143-µm length. Infection with T7Δ0.4 resulted in cells averaging 2.200 rings with a length of 3.513 µm, which closely resembles control measurements and suggests the host can divide normally with the mutant, so gp0.4 plays a role in blocking cell division *via* FtsZ inhibition.

**Figure 7:**
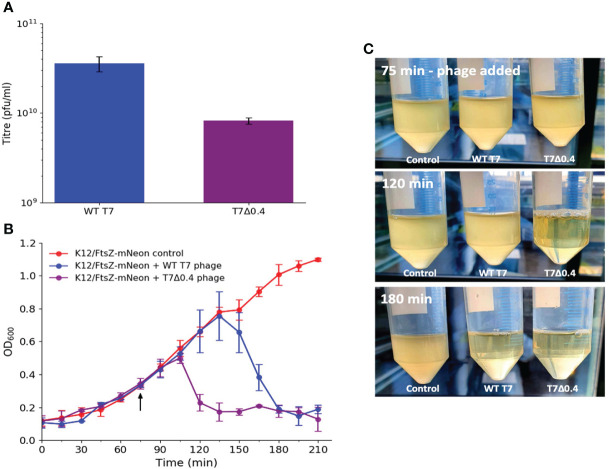
T7Δ0.4 mutant experiments showing that gp0.4 delays lysis and increases progeny. **(A)** WT T7 and T7Δ0.4 phages were propagated and titred by plaque assays in triplicate. Averages plotted on a log scale with error bars showing one standard deviation of uncertainty. **(B)** Growth curves were performed on cultures of K12/FtsZ-mNeon only, K12/FtsZ-mNeon and WT T7, K12/FtsZ-mNeon, and T7Δ0.4 phage. Average readings across replicates at 15-min time intervals were plotted with error bars showing one standard deviation unit of uncertainty. Arrow shows when phage was added. **(C)** Photo time series of growth curves with images of cultures taken upon phage addition at 75 min, at 120 min showing T7Δ0.4 lysis, and 180 min showing WT T7 lysis.

We then investigated why phages may have evolved these peptides, and it has been shown that WT phage progeny is higher than T7Δ0.4 progeny *via* PCR (Kiro et al., 2013), so we further verified this by performing plaque assays to titre phage lysate grown in the presence and absence of gp0.4 (**Figure 7A**). There was nearly a 10-fold decrease in titre with the T7Δ0.4 mutant, supporting previous PCR findings and suggesting gp0.4 plays a role in increasing progeny numbers, potentially by enabling the formation of long undivided cell factories. Growth curves were done to compare phage infection and cell lysis of WT T7 and T7Δ0.4 (**Figures 7B** **, C**), and we found that T7Δ0.4 lysed host cells after 45 min, whereas the WT T7 took 105 min, suggesting that the gp0.4 peptide has a role in delaying host cell lysis by approximately 1 h (43%).

#### 3.5.2 Phage inhibition of FtsZ is limited in the stationary phase and under bacteriostatic conditions

**Figure 8:**
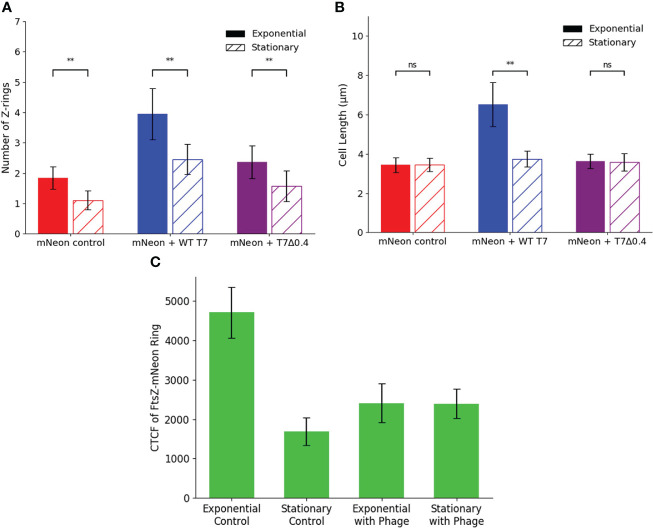
Stationary phase microscopy quantification of WT T7 and T7Δ0.4 infection. Quantification of **(A)** Z-ring number and **(B)** cell length for 75 K12/FtsZ-mNeon bacterial cells infected with T7-mCherry or T7Δ0.4 phage during the stationary phase of growth, along with a no-phage control, compared to exponential phase data. Tukey’s tests were performed, and the calculated probability values (p-values) are displayed as p < 0.05 (*), p < 0.01 (**), and not statistically significant p > 0.05 (ns). **(C)** Exponential phase control, stationary phase control, exponential phase with T7 phage, and stationary phase with T7 phage cultures of *Escherichia coli* K12/FtsZ-mNeon imaged on agarose pads, and the corrected total cell fluorescence (CTCF) of 50 Z-rings per sample was averaged and plotted. Error bars show one standard deviation of uncertainty ns, not significant.

We then wanted to apply the same phages to the model system during the stationary phase of cell growth to test the hypothesis that phage proteins need hosts that are actively dividing and producing an FtsZ target to inhibit FtsZ and block cell division. hCMECs were infected with K12/FtsZ-mNeon cells from overnight cultures to give cells in the stationary phase, followed by incubation with either T7-mCherry or T7Δ0.4 phage, along with a no-phage control. Bacterial cells were quantified and plotted alongside the exponential phase quantification for comparison (**Figures 8A** **, B**).

The average number of Z-rings in the stationary phase control was 1.160, unlike the exponential control of 1.920 rings per cell, suggesting no movement of FtsZ to confirm cells are not dividing. When the phages are applied, there is an increase in ring number for both phages compared to the control, but there are fewer rings produced compared to the exponential phase, suggesting some inhibition is taking place, but it is limited in the stationary phase. There is no change in length upon either phage addition in the stationary phase, so cells are unable to become filamentous. These data are consistent with the hypothesis that T7 inhibition of bacterial cell growth requires the presence of a functional FtsZ engaged in cell division. This was further confirmed by performing growth curves with bacteriostatic concentrations of chloramphenicol (**Figure S13**), which showed the phage was unable to lyse static host cells. Finally, we investigated whether the concentration of FtsZ changed in the stationary phase and upon phage infection by live-cell imaging calculation of corrected total cell fluorescence (CTCF) (**Figure 8C** and **Figure S14**). FtsZ-mNeon imaging shows a 2.8-fold decrease in the intensity per Z-ring in the stationary phase compared to exponential. Interestingly, upon phage addition to exponential culture, the average intensity per ring almost halves, potentially due to FtsZ being spread through the elongated cell.

## 4 Discussion

In this study, we used an *in vitro* meningitis model (Moller-Olsen et al., 2020) to investigate the effect of phage on bacterial FtsZ localisation during treatment, using hCMEC human brain cell cultures infected with *E. coli* EV36/FtsZ-mCherry or K12/FtsZ-mNeon strains and initially observed FtsZ localising to the cell midbody as a single ring. It has been previously shown that the bacteriophage λ Kil peptide can block FtsZ polymerisation and Z-ring formation, leading to a filamentous cell phenotype as a result of cells being unable to complete division (Haeusser et al., 2014). Here we confirm this phenotype and further show for the first time that Kil also leads to the formation of multiple Z-rings in the filamentous cell.

We have engineered a fluorescent T7-mCherry phage by homologous recombination, which specifically targets K12 strains of *E. coli*. This has allowed us to observe the invasion of hCMEC human brain cells by this phage and show that during T7 infection of K12, there is a mislocalisation of FtsZ into multiple rings along a filamentous cell that is unable to divide, suggesting that T7 phage inhibits FtsZ to block cell division. These multiple rings may represent attempts at cell division, where the inhibitor has stalled cytokinesis, creating multiple midbodies since the new upcoming cells are unable to separate. The filamentation seen may be due to MinC inhibition, since we show a MinC mutant strain becomes filamentous in normal growth conditions.

We have also shown that actively dividing cells are needed for phage inhibition of FtsZ, as inhibition is limited in the stationary phase. Since phage is still able to bind and infect these cells, we hypothesise that the decrease in average cell length and ring number is due to the small number of cells that are still dividing at a slower rate, leading to less of an effect seen from gp0.4 or any other inhibitor.

The T7 phage inhibition of FtsZ is likely to have a similar effect on other divisome proteins since proteins such as ZipA and FtsA have been shown to be recruited to the FtsZ ring site, so it is likely they will also mislocalise away from the midbody as new Z-rings form along the cell (Haeusser et al., 2014; Chen et al., 2017). Here we present that the ZapA protein is needed for phage inhibition of FtsZ by showing a phenotype recovery with a ZapA mutant strain, and we show that FtsI protein is also mislocalised upon phage infection. We propose that this effect is specific to the divisome due to the phenotype seen; for example, if the elangosome (Szwedziak and Löwe, 2013) protein MreB was inhibited and mislocalised, we would expect to see lemon-shaped cells (Molshanski-Mor et al., 2014) rather than elongation; therefore, these other proteins appear to be functioning as expected.

A recent study has shown that the T7 phage peptide gp0.4 directly inhibits FtsZ by binding the protein and preventing Z-ring assembly, giving *E. coli* cells an elongated phenotype under light microscopy, to enhance the phage competitive ability (Kiro et al., 2013). In this study, we further investigated how and why phage proteins might target FtsZ, using the T7Δ0.4 mutant to confirm that the T7 peptide gp0.4 is responsible for inhibiting FtsZ by showing a reversal in phenotype to normal single central ring growth compared to the filamentous multi-ring phenotype seen with the wild type. Plaque assays were performed to titre propagated T7Δ0.4 and wild-type T7 phages and found a 10-fold decrease in titre with the mutant, suggesting that the gp0.4 peptide assists in increasing phage progeny, confirming what was previously shown by Kiro et al. using PCR analysis (Kiro et al., 2013), to give the phage a competitive advantage by producing more progeny per burst cycle.

Here we present for the first time that gp0.4 delays host cell lysis, showing that the mutant T7Δ0.4 phage lysed bacterial culture approximately 60 min before the wild-type T7 containing lysed gp0.4. The Kil peptide has similarly been reported to delay lysis by 30% (Haeusser et al., 2014), so it seems that both these phage peptides targeting FtsZ are able to cause this delay. Lysis is initiated when cell signals such as lysins reach a certain threshold concentration towards the end of the infection cycle (Cahill and Young, 2019). Here we hypothesise that the delay in lysis is due to the cell filamentation increasing the volume of the infected cell, and therefore, it is take longer for a similar number of signals to reach the threshold concentration. This could also explain why the reduced titre seen for the mutant since lysis occurs faster, so the host cell bursts prematurely and the phage does not fully exploit the entire resources.

We report for the first time that the K1F phage is also able to target FtsZ and leads to a multi-ringed filamentous phenotype to block cell division, suggesting multiple phages have evolved this strategy.

K1F is a natural T7-like phage (Scholl and Merril, 2005). Interestingly, it lacks the 0.4 genes found in T7 but still shows the same phenotype, so it has evolved another peptide with the same function, which is currently unknown. Further studies on K1F phage to find this inhibitory peptide could lead to the discovery of a new antimicrobial compound. A BLAST search on the National Center for Biotechnology Information (NCBI) database showed that the K1F phage genome had no sequence similarity to the *kil* or *gp0.4* genes, so the inhibitory peptide is likely to be novel from these.

We observed that after about 75 min into normal growth the K12 bacterial cells seem to form two polar Z rings instead of the characteristic single midbody ring in the absence of phage infection. The location of the Z-ring is restricted by regulatory systems such as Min in *E. coli* (Shen and Lutkenhaus, 2011), so two Z-rings may demonstrate a time within the cell in which the Min system has not started to discriminate against FtsZ at the poles (Hu et al., 1999) and has also been previously shown that competition between FtsZ and the Min system could be the cause for these polar sites (Hu et al., 1999). We further show that a knockout mutant lacking the MinC protein displays a filamentous phenotype under normal growth conditions.

In conclusion, we have shown that FtsZ assembles as a single ring at the cell midbody under normal bacterial growth conditions during infection of human cells and shown that this localisation is disrupted to give a filamentous phenotype with multiple Z-rings along the cell body *via* phage peptides such as Kil, gp0.4, and an unknown K1F phage protein during phage infection. This inhibition may function to delay host lysis and increase phage progeny numbers and occurs during the exponential phase of bacterial growth when cells are dividing. This furthers our understanding of the mechanism that phages use to control their bacterial host, allowing them to be considered future antimicrobials.

## Data availability statement

The original contributions presented in the study are included in the article/**supplementary material**. Further inquiries can be directed to the corresponding author.

## Author contributions

GD designed and performed experiments and wrote and revised the manuscript. IK performed experiments. UQ revised the manuscript. DR advised on experimental design and setup and revised the manuscript. AS conceived the idea, designed the experiments, supervised the study, and revised the manuscript. All authors contributed to the article and approved the submitted version.

## Funding

This work was funded by the Biotechnology and Biological Sciences Research Council (BBSRC) Future Leader Fellowship (ref. BB/N011872/1) to A.P.S., and the BBSRC and University of Warwick funded Midlands Integrative Biosciences Training Partnership (MIBTP) to G.K.D. I.K was supported by the MBio Masters programme at the University of Warwick.

## Acknowledgments

The authors would like to thank Dr Harold Erickson (Duke University School of Medicine) for providing the K12/FtsZ-mNeon strain, Dr Kenn Gerdes (University of Copenhagen) for providing the FtsZ-mCherry plasmid, and Dr William Margolin (University of Texas McGovern Medical School) for providing the Kil peptide plasmid. They would also like to thank Dr Ian Hands-Portman for confocal microscopy training and CLEM imaging, Dr Saskia Bakker at the Warwick Advanced Bioimaging Research Technology Platform for electron microscopy training, and John Moat and Abby Henney at the Warwick Antimicrobial Screening Facility for identifying minimum inhibitory concentrations.

## Conflict of interest

The authors declare that the research was conducted in the absence of any commercial or financial relationships that could be construed as a potential conflict of interest.

## Publisher’s note

All claims expressed in this article are solely those of the authors and do not necessarily represent those of their affiliated organizations, or those of the publisher, the editors and the reviewers. Any product that may be evaluated in this article, or claim that may be made by its manufacturer, is not guaranteed or endorsed by the publisher.
