# Supplementary material for: Investigating the effect of bacteriophages on bacterial FtsZ localisation
Source: Front Cell Infect Microbiol. 2022 Jul 29;12:863712. doi: 10.3389/fcimb.2022.863712 (PMC9372555; doi:10.3389/fcimb.2022.863712)
Supplement: Supplementary file 1 [file DataSheet_1.pdf]

Supplementary material

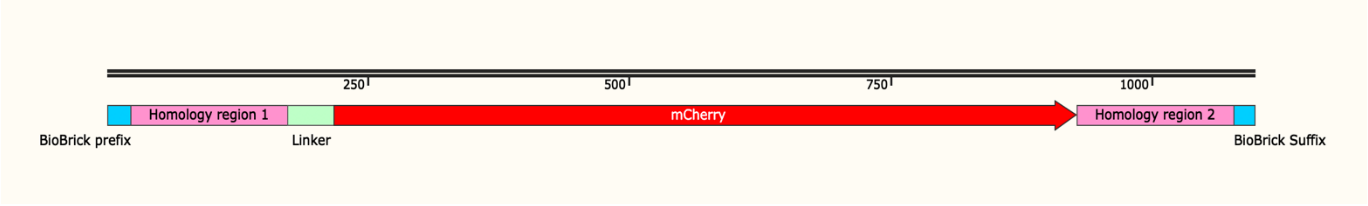

Figure S1: gBlock insert designed to make fluorescent T7 phage. A gBlock was designed to create a recombinant T7-mCherry phage. The BioBrick regions at the ends shown in blue contain restriction sites to allow cloning into a vector. Homology regions shown in pink contain 150 bp of DNA before and after the stop codon of the minor capsid protein. The mCherry gene is shown in red and the linker region is shown in green.

Primer	Sequence
AG005	GAATTCGCGGCCGCTTCTAGA
AG006	CTGCAGCGGCCGCTACTAGTA
mCherry forward	ATGGTGAGCAAGGGCGAG
mCherry reverse	CTACTTGTACAGCTCGTCCATGC

Table S1: Primers. Primers used in the engineering of T7-mCherry phage

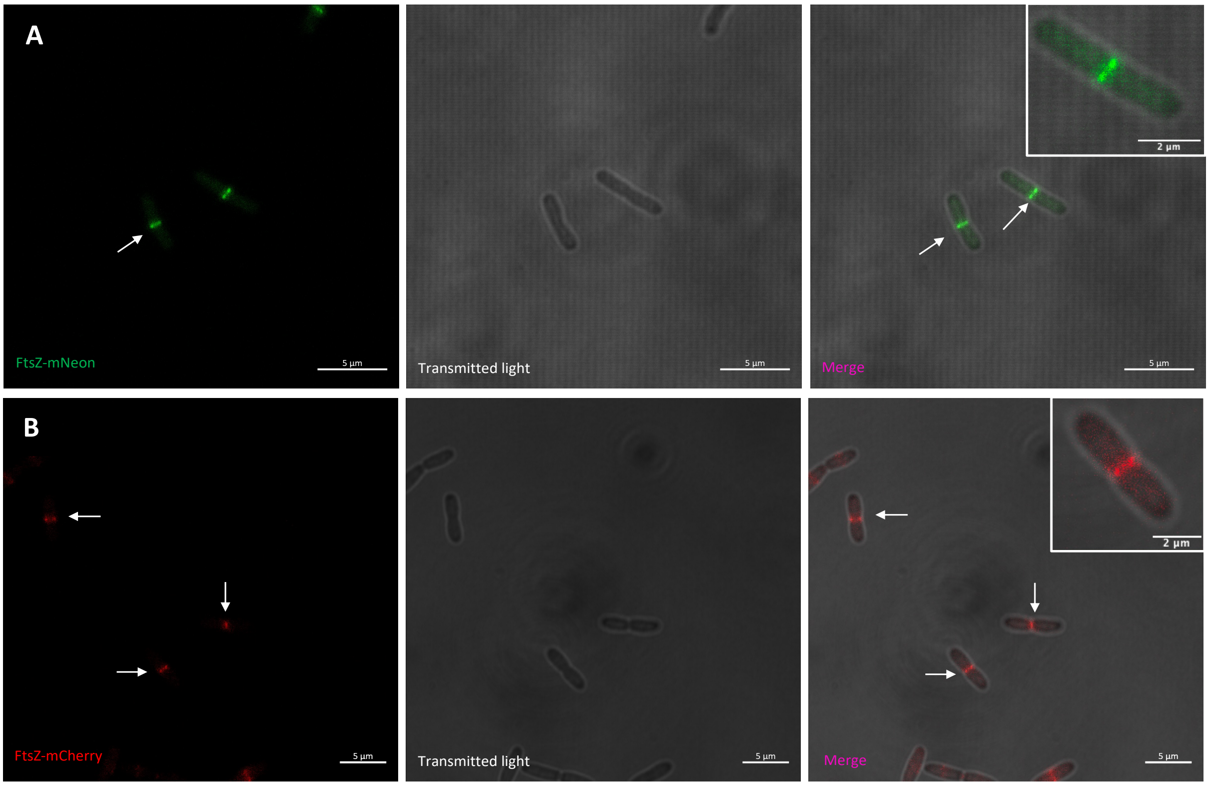

Figure S2: Visualisation of the FtsZ Z-ring in normal growth conditions by confocal microscopy. (A) Fluorescent images showing live K12/FtsZ-mNeon cells on agarose pads. **(B)** Live EV36/FtsZ-mCherry cells on agarose pads. FtsZ-mNeon is shown in green and FtsZ-mCherry is shown in red.

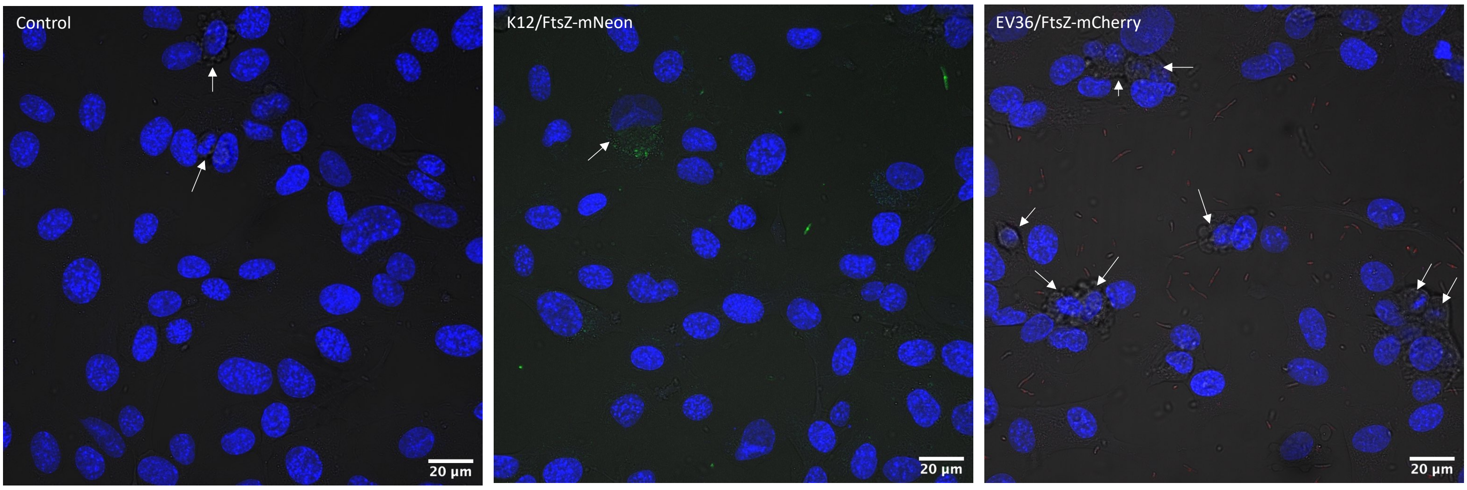

Figure S3: Human cell death by extracellular bacterial strains. HCMEC human brain cells were infected with the K12/FtsZ-mNeon and EV36/FtsZ-mCherry strains live, along with a no bacteria control, and numbers of dying human cells were counted and the percentage cell death was quantified as follows: 4.8% hCMEC cell death in control conditions, 5.3% hCMEC cell death following K12 infection, and 12.1% hCMEC cell death following EV36 infection. Cell outlines are shown by transmitted light and DAPI stain was used to show DNA in blue. Arrows are pointing to dying human cells. For each condition the experiments were repeated in triplicate and a minimum of 500 human cells per condition were counted.

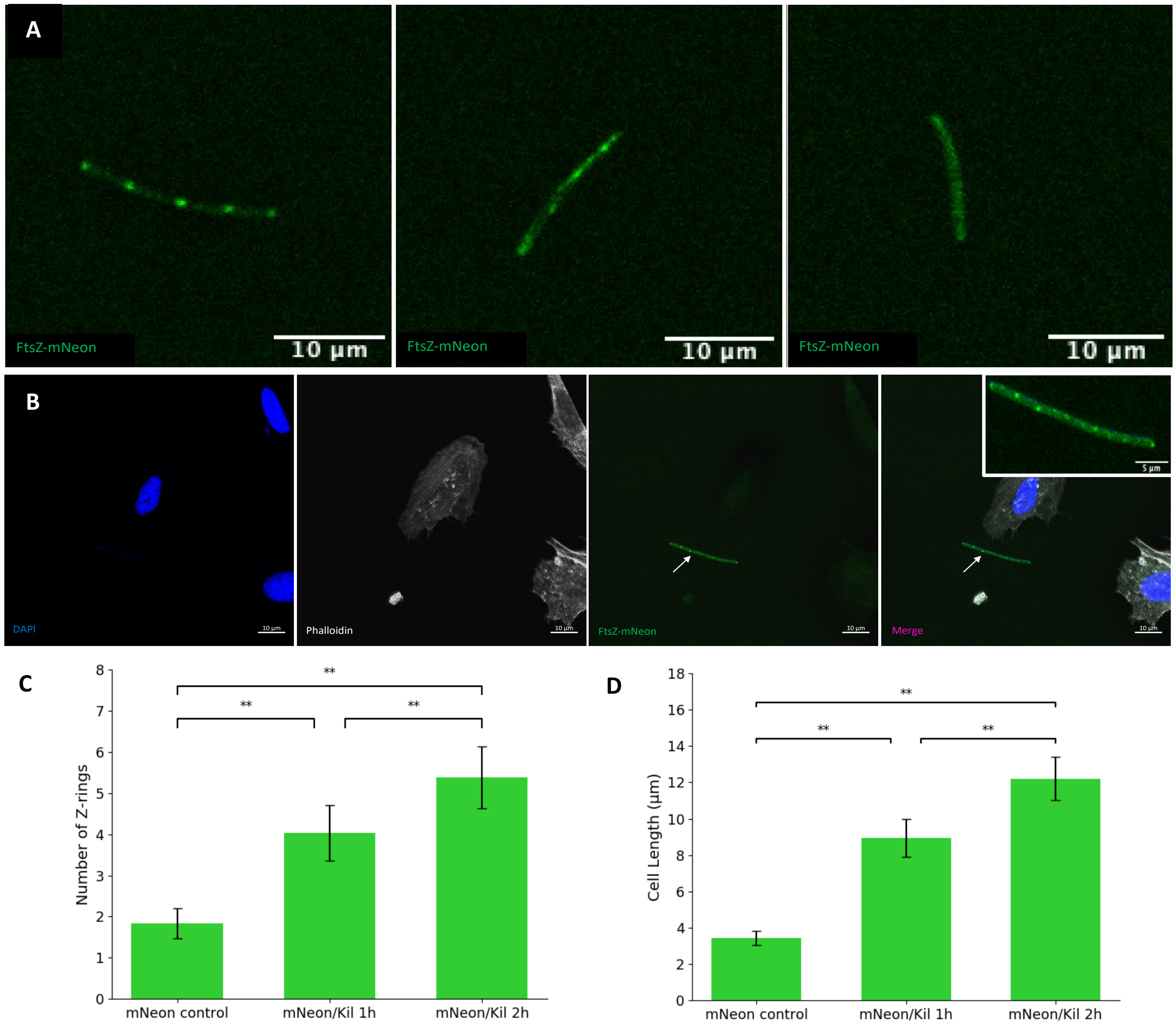

Figure S4: Kil peptide inhibition of *E. coli* FtsZ. (A) Different phenotypes of K12/FtsZ-mNeon/Kil. Left image shows multiple distinct Z-rings, right images shows diffuse spread and middle images shows an intermediate phenotype. **(B)** K12/FtsZ-mNeon/Kil cell fixed in human cell infection. Arrow pointing to filamentous bacterial cells. Images taken one 2 hours after plasmid induction. DAPI stain is shown in blue, phalloidin stain is shown in grey and FtsZ-mNeon is shown in green. **(C-D)** Quantification of Z ring number and cell length for 75 K12/FtsZ-mNeon/Kil bacterial cells under the following conditions: control, 1 hour plasmid induction, 2 hour plasmid induction. Mean averages plotted with error bars showing one standard deviation of uncertainty. Tukey's tests were performed and the calculated probability values (p-values) are displayed as $p \leq 0.05$ (*), $p \leq 0.01$ (**), and not statistically significant $p \geq 0.05$ (ns).

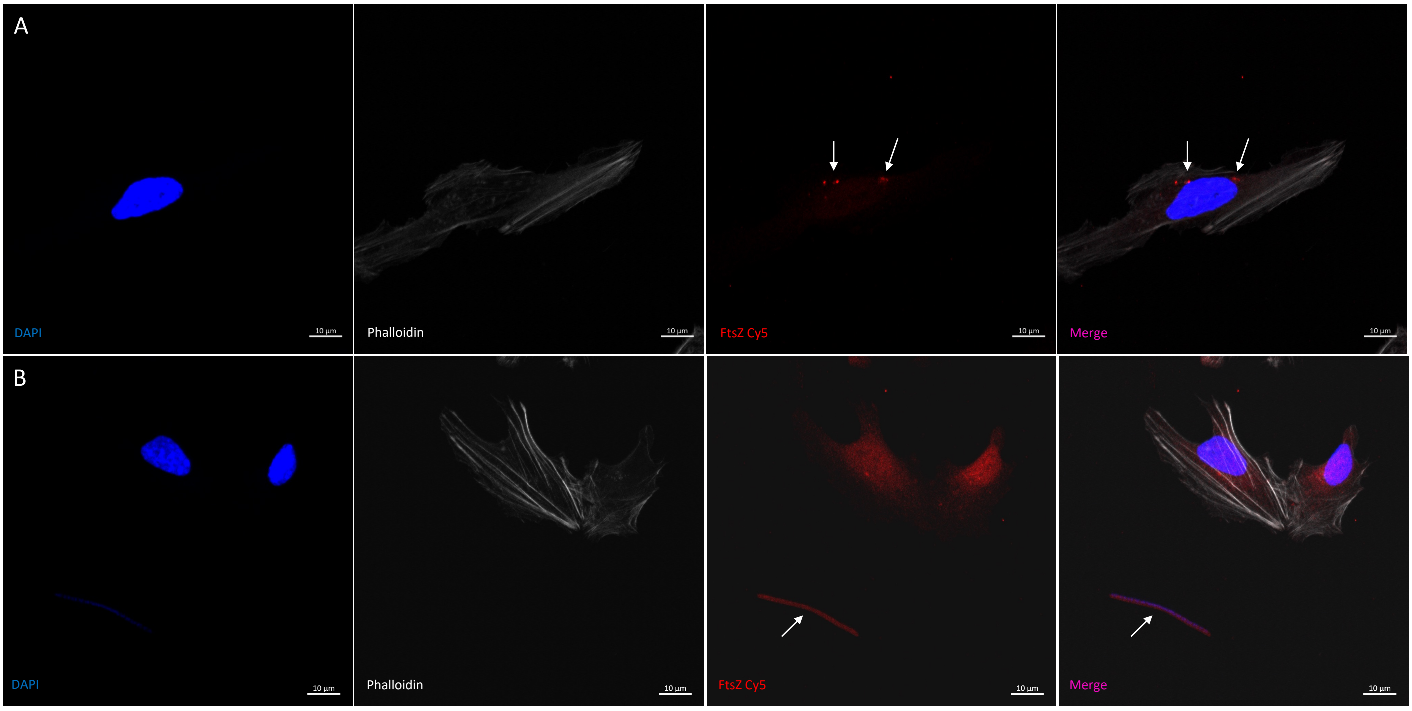

Figure S5: Kil inhibition of E. coli EV36 FtsZ. EV36/Kil cell fixed in human cell infection with anti-Prokaryotic Cell Division GTPase (FtsZ) antibody staining**. (A)** Control with no Kil induction (**B)** Elongated cell after Kil induction

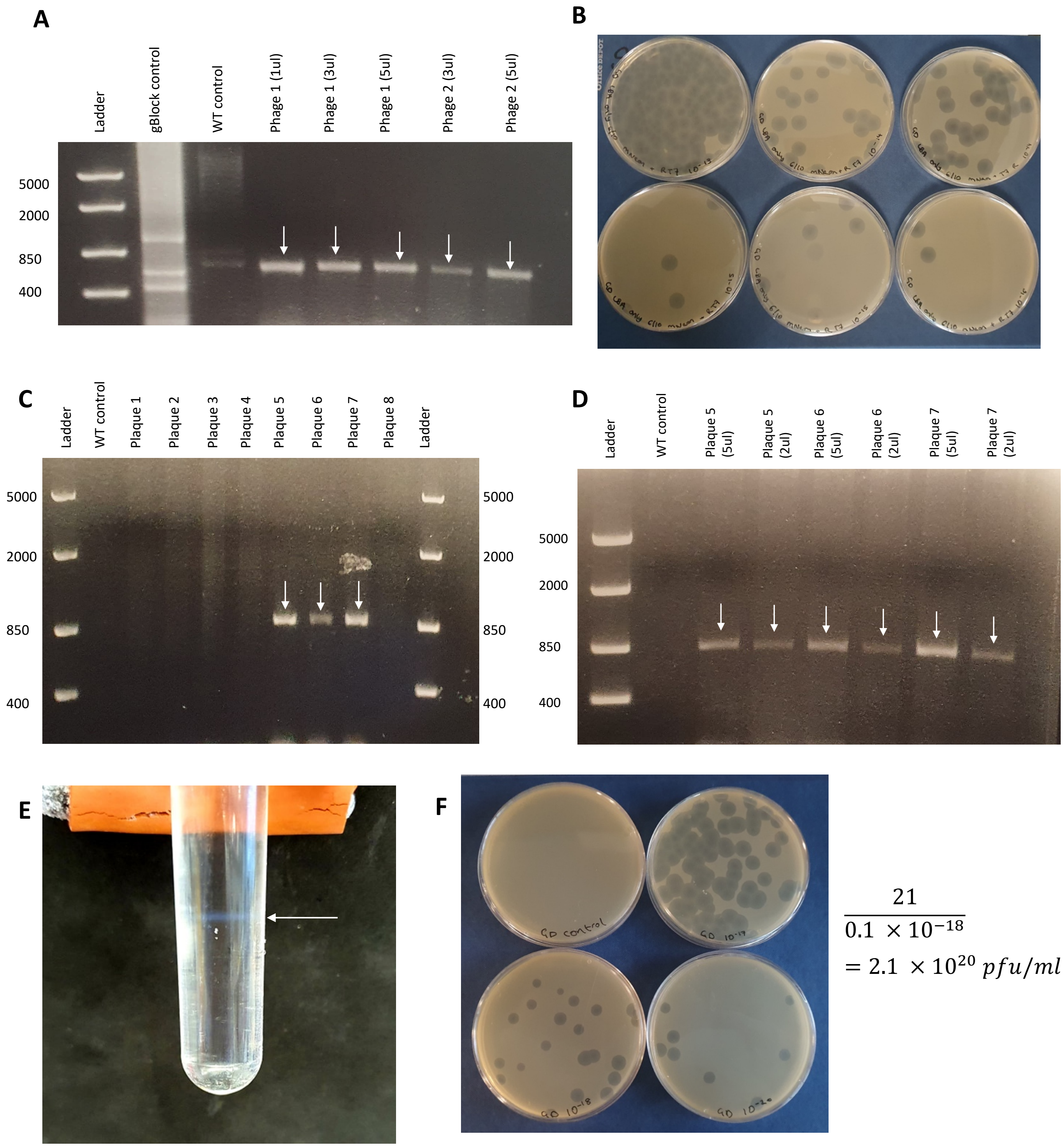

Figure S6: Screening for recombinant phage after homologous recombination
(A) Phage culture screening round 1 PCR after homologous recombination
(B and C) Plaque assay of positive culture and screening of individual plaques
(D) Positive plaques propagated and further PCR test on liquid culture
(E) CsCl purification band of positive T7-mCherry culture
(F) Plaque assay to titre the recombinant phage

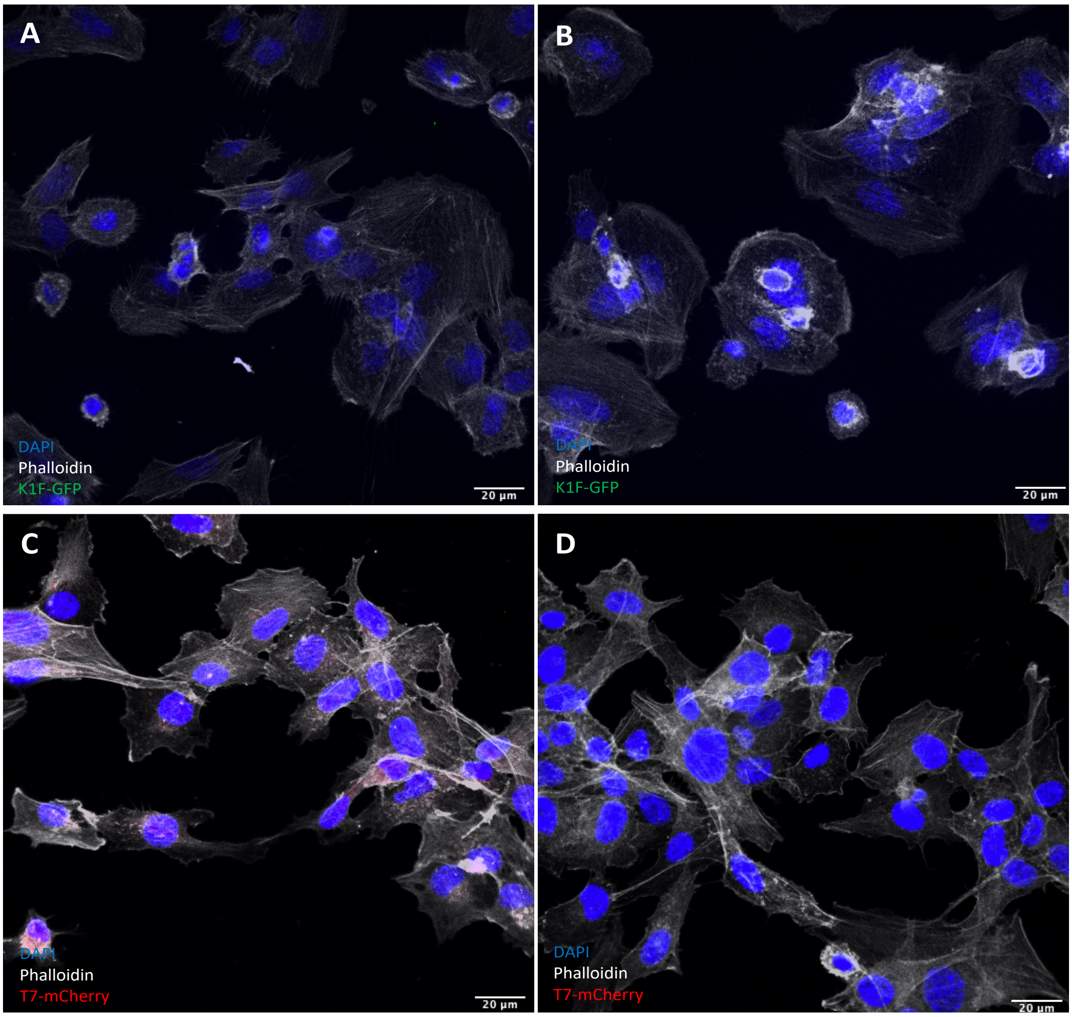

Figure S7: No-phage controls for experiments visualising phages in human cells. **(A-B)** No phage control samples for visualisation of K1F-GFP phage in hCMEC, showing no contamination or background fluorescence inside the human cells**. (C-D)** No phage control samples for visualisation of T7-mCherry phage in hCMEC, showing no contamination or background fluorescence inside the human cells. DAPI stain is shown in blue, phalloidin in grey, K1F-GFP in green and T7-mCherry in red.

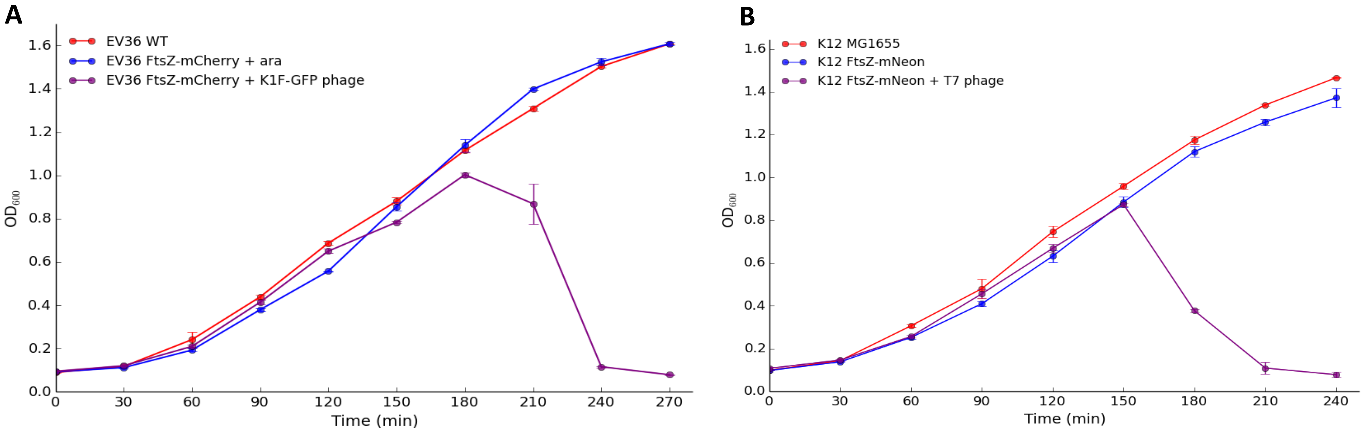

Figure S8: Growth curves. (A) Growth curves for EV36, EV36/FtsZ-mCherry and EV36/FtsZ-mCherry with K1F-GFP phage. OD600 readings were taken every 30 minutes and phage was added after OD600 passed 0.3 at 90 minutes **(B)** Growth curves for MG1655, K12/FtsZ-mNeon and K12/FtsZ-mNeon with T7 phage. OD600 readings taken every 30 minutes and phage was added after OD600 passed 0.3 at 90 minutes.

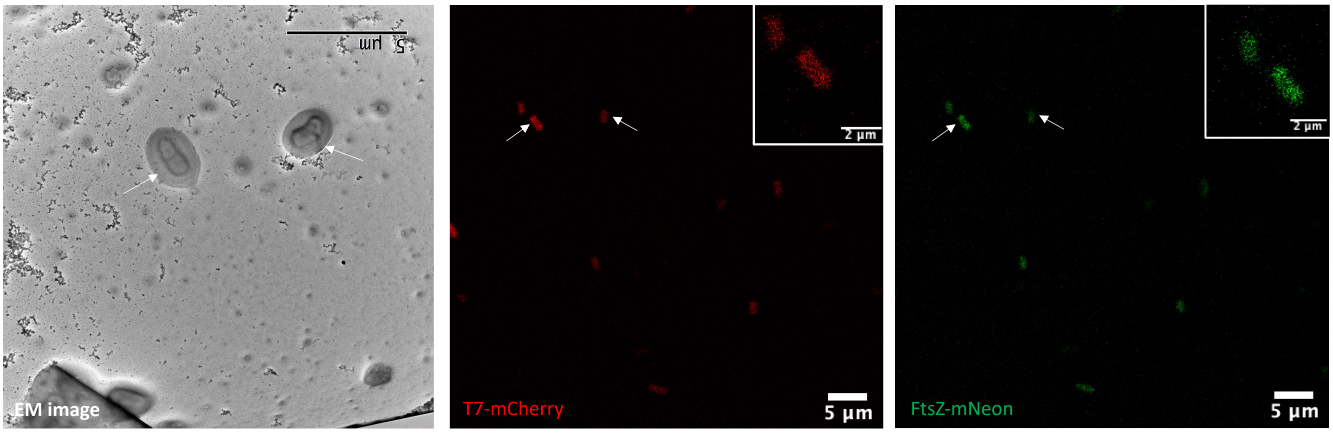

Figure S9: Correlative light and electron microscopy. Correlative light and electron microscopy (CLEM) images of *E. coli* K12/FtsZ-mNeon after 60 minutes of T7 infection. Left panel shows EM image, middle shows confocal T7-mCherry channel and right shows confocal mNeon channel.

	WT T7 titres (pfu/ml)	T7Δ0.4 titres (pfu/ml)
Replicate 1	4×10^{10}	8.36×10^{9}
Replicate 2	4.13×10^{10}	7.3×10^{9}
Replicate 3	2.6×10^{10}	9×10^{9}

Table S2: Table of titres from plaque assays for WT and T7 mutant phages

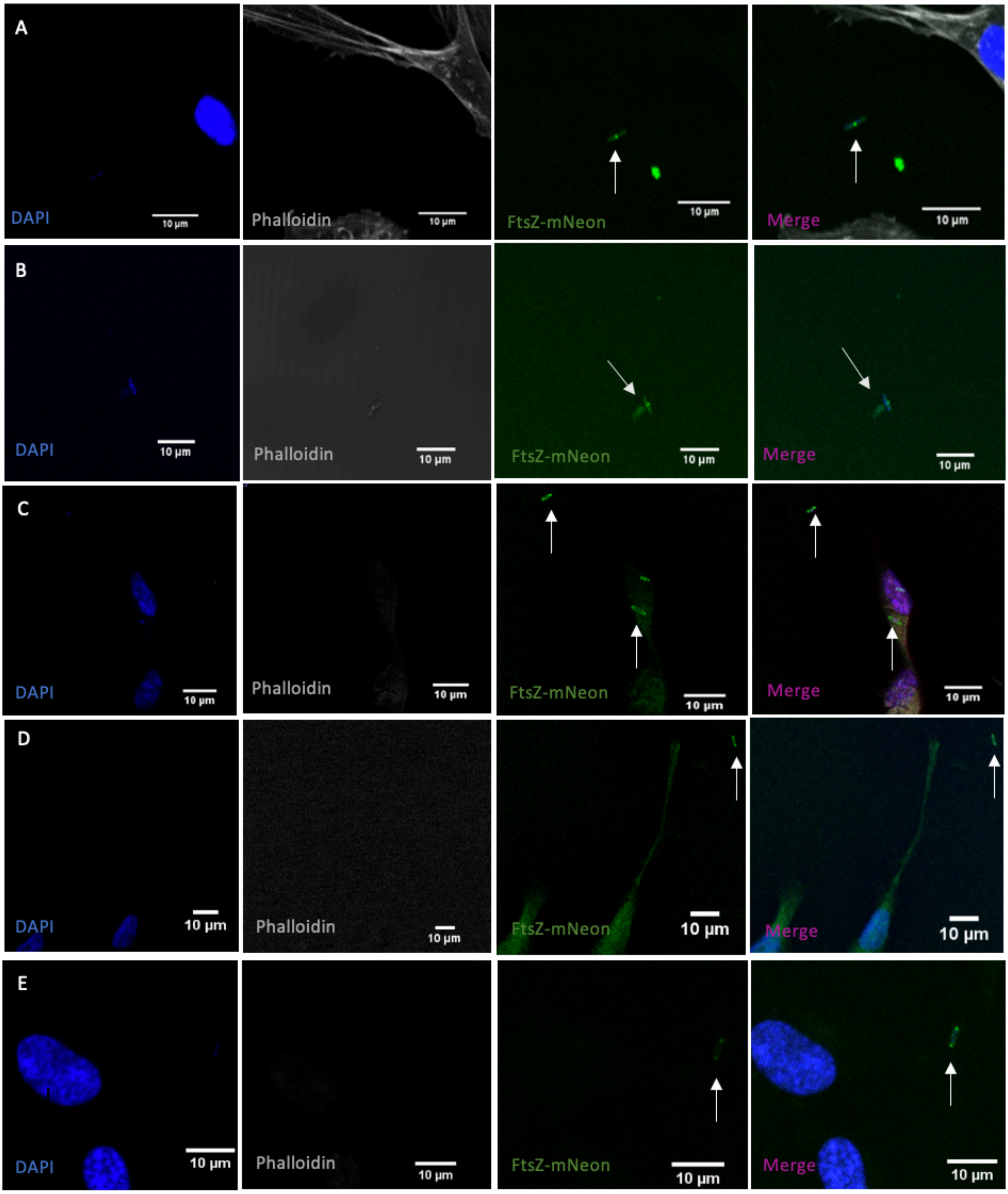

Figure S10. Control (no phage) time series of K12/FtsZ-mNeon in hCMEC cells. (A) 45 minutes incubation of K12/FtsZ-mNeon **(B)** 60 minutes incubation of K12/FtsZ-mNeon **(C)** 75 minutes incubation of K12/FtsZ-mNeon **(D)** 105 minutes incubation of K12/FtsZ-mNeon **(E)** 120 minutes incubation with FtsZ-mNeon.

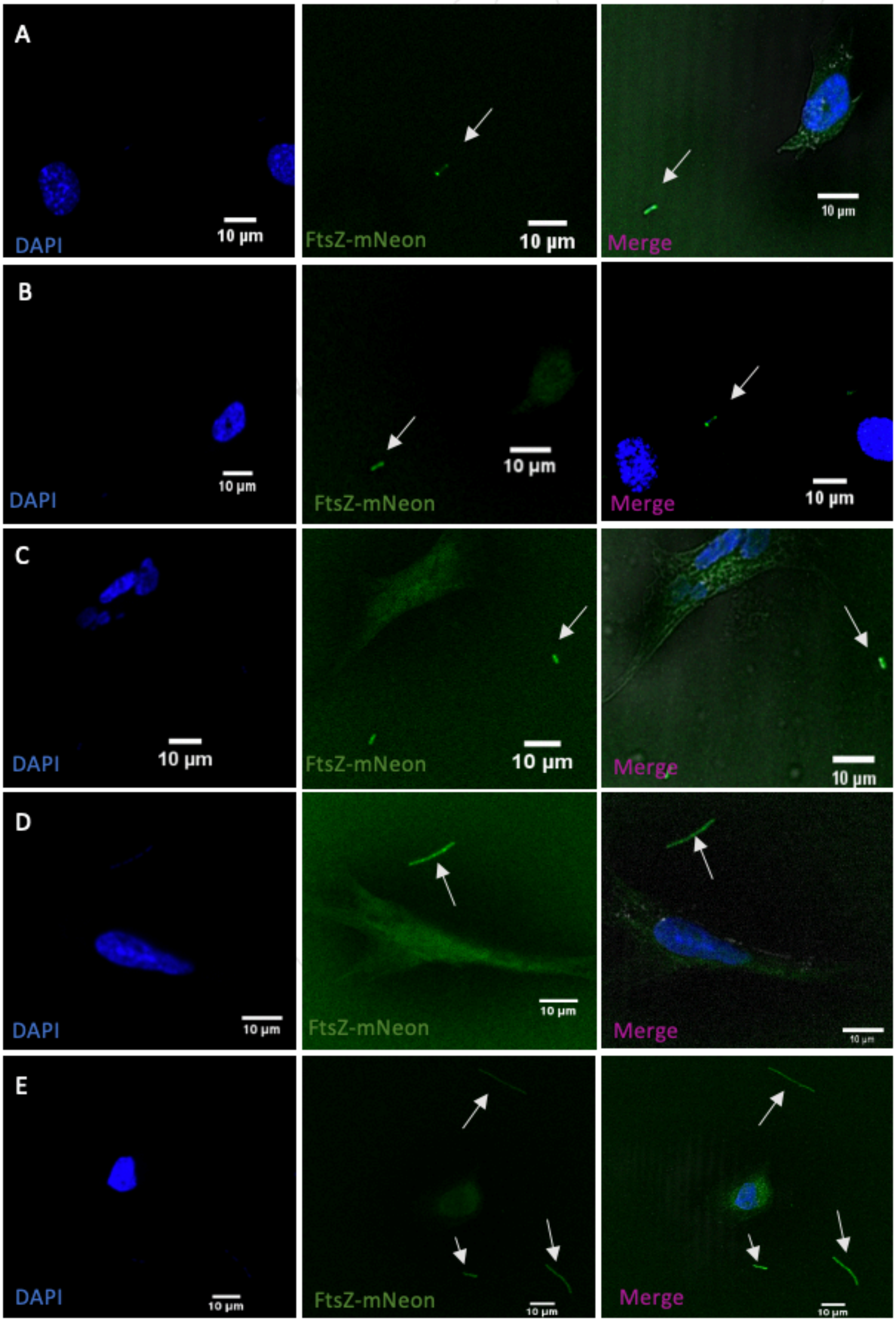

Figure S11. Time series of K12/FtsZ-mNeon infected with T7-mCherry in hCMEC cells. (A) 0 minutes incubation of K12/FtsZ-mNeon with T7-mCherry **(B)** 30 minutes incubation of K12/FtsZ-mNeon with T7-mCherry **(C)** 45 minutes incubation of K12/FtsZ-mNeon with T7-mCherry **(D)** 60 minutes incubation of K12/FtsZ-mNeon with T7-mCherry **(E)** 75 minutes incubation of K12/FtsZ-mNeon with T7-mCherry.

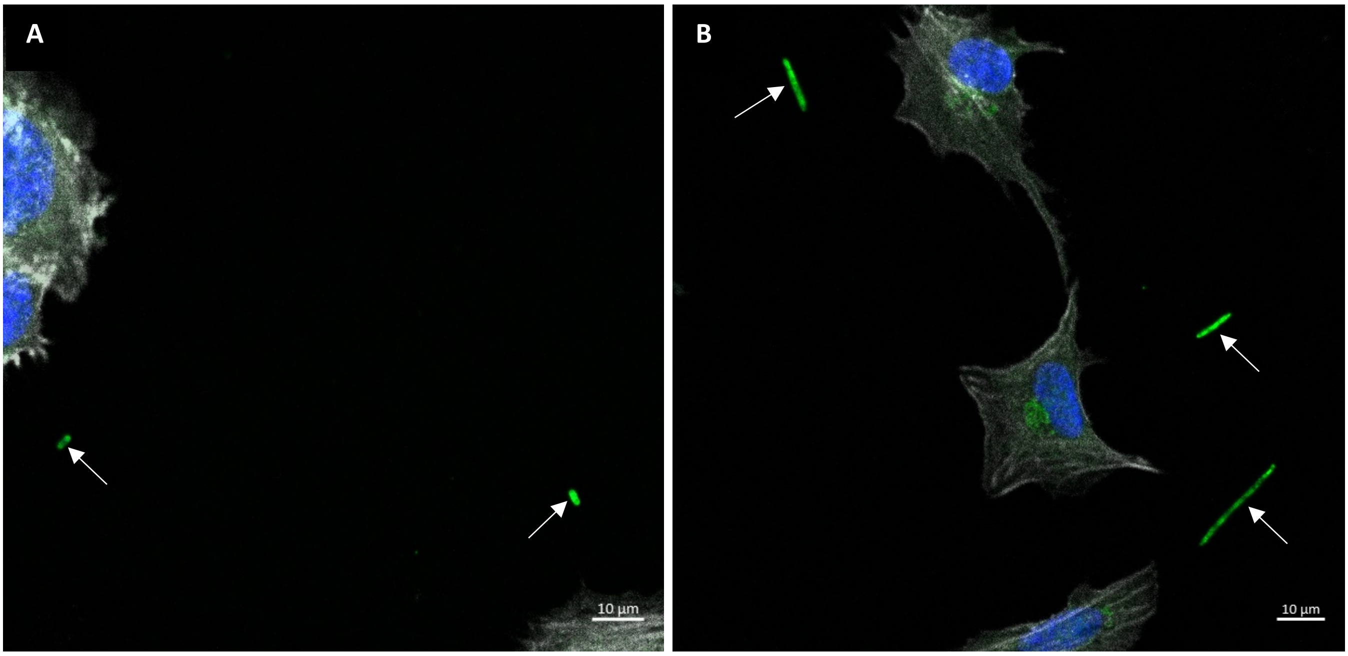

Figure S12: Mutated MinC leads to cell filamentation (A) Confocal microscopy image of K12 control cells after FtsZ antibody staining. **(B)** Confocal microscopy image of K12ΔminC cells after FtsZ antibody staining. DAPI stain is shown in blue, phalloidin in grey, and FtsZ in green. Arrows point to cells of interest.

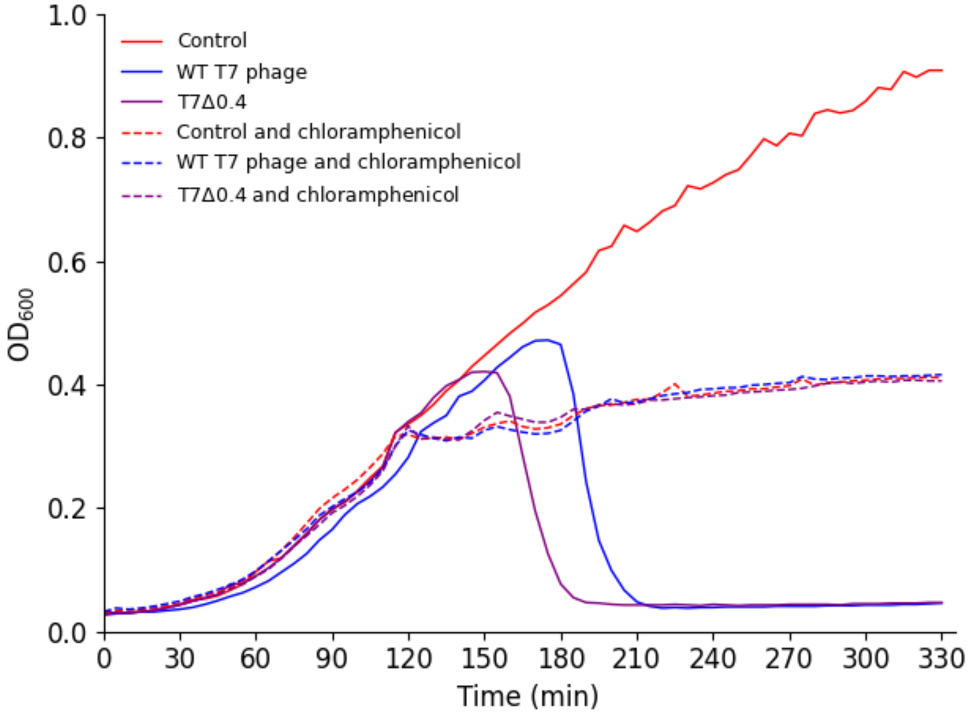

Figure S13: Growth curve showing the effect of bacteriostatic antibiotics on host cell lysis. Growth curves for K12/FtsZ-mNeon with WT T7 phage and T7Δ0.4 phage, alongside a no phage control (solid lines), and curves for K12/FtsZ-mNeon with WT T7 phage and T7Δ0.4 phage, alongside a no phage control with a bacteriostatic concentration of chloramphenicol added to each culture (dashed lines). OD600 readings taken every 5 minutes, and phage and chloramphenicol was added when needed after OD600 passed 0.3 at 90 minutes.

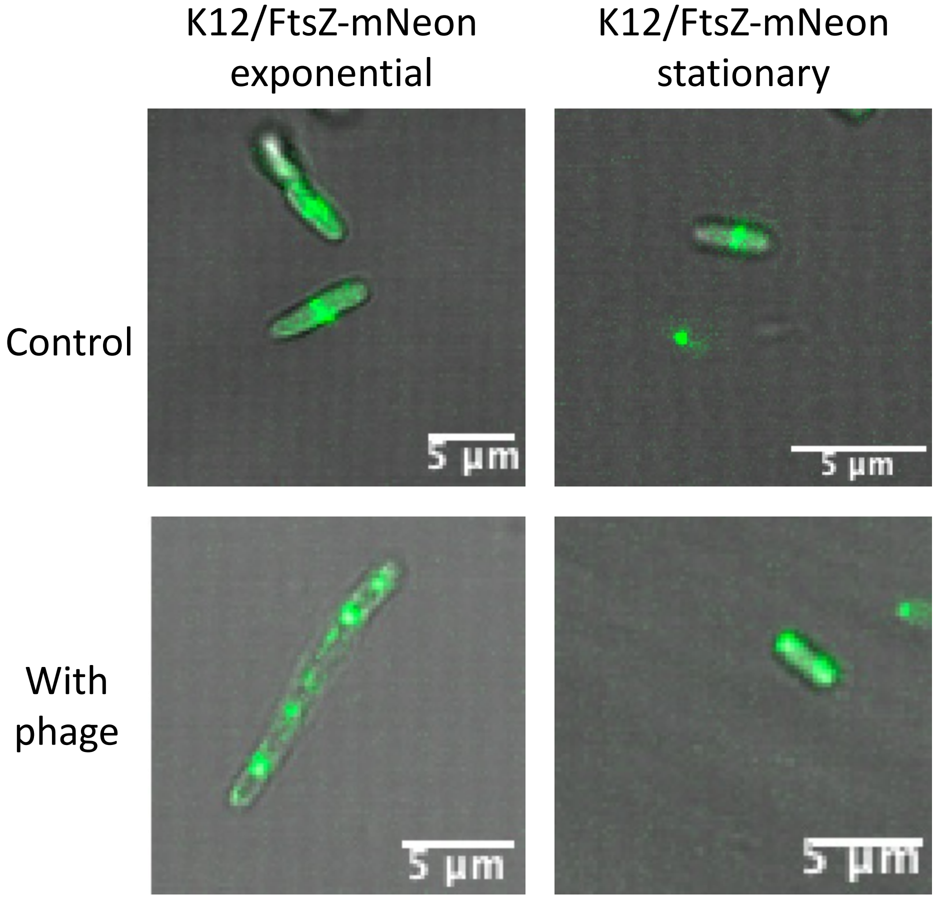

Figure S14: Representative images used for calculated total cell fluorescence (CTCF). Cultures of *E. coli* K12/FtsZ-mNeon in the exponential phase, K12/FtsZ-mNeon in the stationary phase, K12/ FtsZ-mNeon and T7 phage in the exponential phase, and K12/ FtsZ-mNeon and T7 phage in the stationary phase were grown and imaged on triplicate agarose pads. 50 cells per condition were quantified for their CTCF using fluorescence intensity measurements calculated on FiJi.
